# The European Program for Prevention (EPP) – Implementing Proven Preventing Measures Now!

**DOI:** 10.5334/gh.1491

**Published:** 2025-11-14

**Authors:** Maciej Banach, Zlatko Fras, Dan Gaita, Ivan Pecin, Gani Bajraktari, Bojko Bjelakovic, Ibadete Bytyci, Richard Ceska, Dragan Djuric, Robert J. Gil, Jacek Jozwiak, Raimondas Kubilius, Gustavs Latkovskis, Olena Mitchenko, Gyorgy Paragh, Daniel Pella, Zaneta Petrulioniene, Arman Postadzhiyan, Anca Pantea Stoian, Piotr Szymański, Margus Viigimaa, Dragos Vinereanu, Branislav Vohnout, Michal Vrablik, Zeljko Reiner, For The International Lipid Expert Panel (ILEP)

**Affiliations:** 1 Faculty of Medicine, John Paul II Catholic University of Lublin, Lublin, Poland; 2 Department of Preventive Cardiology and Lipidology, Medical University of Lodz (MUL), Lodz, Poland; 3 Ciccarone Center for the Prevention of Cardiovascular Disease, Johns Hopkins University School of Medicine, Baltimore, MD, USA; 4 Centre for Preventive Cardiology, Department of Vascular Medicine, University Medical Centre Ljubljana, Slovenia; 5 Faculty of Medicine, University of Ljubljana, Slovenia; 6 Institute for Cardiovascular Diseases, University of Medicine and Pharmacy Victor Babes, Research Center IBCVTIM, Timisoara, Romania; 7 Department of Internal Medicine, Division of Metabolic Diseases, University Hospital Center Zagreb, Zagreb, Croatia; 8 Department of Internal Medicine, School of Medicine, University of Zagreb, Zagreb, Croatia; 9 Medical Faculty, University of Prishtina, University Clinical Centre of Kosova, Prishtina, Kosovo; 10 Clinic of Pediatrics, Clinical Center, Nis, Serbia; 11 Medical Faculty, University of Nis, Nis, Serbia; 12 Clinic of Cardiology, University Clinical Centre of Kosovo, Prishtina, Kosovo; 13 Department of Public Health and Clinical Medicine, UmeåUniversity, Umeå, Sweden; 14 3rd Department of Internal Medicine, Center of Preventive Cardiology, University General Hospital, Charles University in Prague, Prague, Czech Republic; 15 Institute of Medical Physiology “Richard Burian”, Faculty of Medicine, University of Belgrade, Belgrade, Serbia; 16 Department of Cardiology, National Medical Institute of the Ministry of Interior and Administration, Warszawa, Poland; 17 Department of Family Medicine and Public Health, Institute of Medical Sciences, University of Opole, Poland; 18 Department of Rehabilitation, Faculty of Nursing, Medical Academy, Lithuanian University of Health Sciences, Kaunas, Lithuania; 19 Latvian Centre of Cardiology, Pauls Stradins Clinical University Hospital, Riga, Latvia; 20 Faculty of Medicine and Life Sciences, University of Latvia, Riga, Latvia; 21 Dyslipidaemia Department, Institute of Cardiology, AMS of Ukraine, Kiev, Ukraine; 22 Division of Metabolism, Department of Internal Medicine, Faculty of Medicine, University of Debrecen, Debrecen, Hungary; 23 2nd Department of Cardiology of the East Slovak Institute of Cardiovascular Diseases, Faculty of Medicine, Pavol Jozef Safarik University in Kosice, Kosice, Slovakia; 24 Institute of Clinical Medicine, Faculty of Medicine, Vilnius University, Vilnius, Lithuania; 25 Cardiology Clinic, UMBAL “Saint Anna”–Sofia, Medical University Sofia, Sofia, Bulgaria; 26 Department of Diabetes, Nutrition and Metabolic Diseases, Carol Davila University of Medicine and Pharmacy, Bucharest, Romania; 27 Clinical Cardiology Centre, National Institute of Medicine of the Ministry of Interior and Administration, Warszawa, Poland; 28 Medical Centre of Postgraduate Education, Warsaw, Poland; 29 North Estonia Medical Centre, Tallinn University of Technology, Tallinn, Estonia; 30 University of Medicine and Pharmacy Carol Davila, Department of Cardiology and Cardiovascular Surgery, Bucharest, Romania; 31 University and Emergency Hospital, Bucharest, Romania; 32 Institute of Nutrition, Faculty of Nursing and Health Professional Studies and Coordination Centre for Familial Hyperlipoproteinemias, Slovak Medical University in Bratislava, Bratislava, Slovakia; 33 Department of Diabetology, Faculty of Medicine, Slovak Medical University, Bratislava, Slovakia

**Keywords:** best practices, cardiovascular diseases, Central and Eastern European countries, European Program for Prevention, healthcare programs, prevention, screening

## Abstract

Cardiovascular diseases (CVDs) remain a leading global cause of mortality and disability, with significant disparities observed across countries. This is particularly true in Central and Eastern Europe (CEE), where populations are primarily at high and very high CVD risk. Highlighting modifiable risk factors underscores the urgent need for effective prevention programs. This paper introduces the European Program for Prevention (EPP), an initiative by the International Lipid Expert Panel (ILEP), designed to address these challenges. The EPP aims to enhance awareness and knowledge of validated preventive healthcare solutions implemented in CEE countries, showcase the region’s potential for innovative strategies, and evaluate the adaptability of successful programs for broader implementation. The EPP strongly supports the EU Cardiovascular Health Plan, as well as initiatives by the World Heart Federation (WHF) and World Health Organization (WHO), by promoting best practices, early detection, integrated prevention frameworks, training, cross-border cooperation, and policy development. It advocates shifting healthcare priorities towards pre-disease prevention, thus reducing reliance on resource-intensive treatments. The program proposes an optimal CVD prevention system that includes mandatory health education, screening programs for familial hypercholesterolemia and universal Lp(a) screening, and comprehensive check-ups, notably integrated, comprehensive care programs. By leveraging existing validated programs and fostering collaboration, the EPP seeks to reduce the burden of CVD, improve outcomes, and promote cardiovascular health across Europe and beyond.

## Worrying Cardiovascular Epidemiological Metrics

The recent results from the Global Burden of Disease (GBD) 2023 study, which included data from 204 countries and territories spanning from 1990 to 2023, confirmed again that cardiovascular diseases (CVDs) are the leading cause of disability-adjusted life years (DALYs) and deaths (1). In 2023, there were 437 million CVD DALYs globally, representing a 1.4-fold increase from the number in 1990. Ischemic heart disease (IHD), ischemic stroke, hypertensive heart disease, and intracerebral hemorrhage were the leading causes of CVD DALYs globally in 2023, with the most severe outcomes in low and low-middle Socio-Demographic Index (SDI) countries (1). The number of CVD deaths increased globally from 13.1 million in 1990 to 19.2 million (17.4 to 20.4 million) in 2023, reflecting an absolute increase of 6.1 million deaths over the last 33 years. The number of prevalent cases of CVD has more than doubled since 1990, reaching 626 million globally in 2023. Importantly, a total of 79.6% of the CVD burden is attributable to modifiable risk factors, with high systolic blood pressure (SBP), dietary risks, high low-density lipoprotein cholesterol (LDL-C), and air pollution being the primary modifiable risks responsible for most of the attributable CVD burden in 2023 (1). Overall, since 1990, changes in exposure to modifiable risk factors have had mixed effects on the CVD burden, with increases in high body mass index (BMI), high fasting plasma glucose (FBG), and low physical activity (PA) contributing to a higher burden, while reductions in tobacco use have mitigated some of these increases (1,2,3,4,5,6). It is important to emphasize that population growth and aging have been the main drivers of the increasing burden since 1990, contributing an additional 128 million and 139 million CVD DALYs to the overall increase in CVD burden, respectively (1).

Significant variations exist in the burden of CVD even among countries with similar levels of development (7). This gap is largely explained by known, modifiable risk factors that are inadequately managed. Therefore, countries need to adopt effective healthcare programs (preferably coordinated or integrated ones) and public health strategies to improve their progress toward reducing the burden of CVD (1,8). This approach will also enable them to measure advancements toward Sustainable Development Goals Target 3.4, which aims to reduce premature mortality from non-communicable diseases by one-third by the year 2030 (1).

This analysis reveals significant health inequalities among Central and Eastern European (CEE) countries, leading to substantial differences in age-standardized CVD DALYs per 100,000 in 2023 (1). The highest rates were reported in Ukraine (8,766.6 per 100,000), Belarus (9,150.8), Bulgaria (7,886.9), Serbia, Moldova, and Romania, while the lowest rates were seen in Slovenia (2,271.9 per 100,000), Czechia (3,702.2), Poland (3,809.3), and Estonia. Despite these lower figures, outcomes remain worse than in Western European countries, except for Slovenia, which has results even better than in Germany (2,770) and Austria (2,585.4). In terms of age-standardized DALY rates for IHD in 2023, Poland (1,900.8), Estonia (1,530.6), and Slovenia (810) achieved the best results within the CEE group. While there have been significant improvements over the past three decades regarding stroke risk, Bulgaria (2,645.8 compared to 574.6 in Slovenia), Serbia (2,442.6), North Macedonia (2,886.2), Montenegro, and Romania still exhibit concerning stroke DALY rates. The age-standardized DALY rates for hypertensive heart disease in 2023 mirrored the troubling stroke results. Rates for peripheral artery disease (PAD) are especially alarming and warrant urgent action (what was already raised in the GBD 2021 analysis (9)), excluding Bosnia and Herzegovina (13.5), Albania (9.6 compared to 79.1 in Belarus and 65.3 in Latvia), North Macedonia (11.9), and Bulgaria (12.6) (1).

The distribution and prevalence of modifiable risk factors in CEE countries largely explain these outcomes, particularly concerning smoking, alcohol use, and low physical activity, with Slovenia still displaying the best results in the region (1). A recently published GBD analysis for Poland (10), in comparison with other countries in the region, confirmed these findings, showing that reductions in deaths from IHD and stroke significantly contributed to improvements in life expectancy in 2023 (from 109,000 and 68,300 deaths due to IHD and stroke in 1990, respectively, to 85,400 and 39,500 in 2023). Smoking and high blood pressure (BP) were the leading risk factors throughout the study period, while alcohol use exhibited the greatest increase (35.2%) in DALY rates between 1990 and 2023. At the same time, risk-attributable, age-standardized DALY rates declined for high BP (from 5,723.8 to 2,053.7) and high body mass index (BMI) (from 2,226.4 to 1,923.4). However, the greatest decreases were observed for particulate matter pollution (77.9%) and high LDL-C (70.9%) (10).

## Why is the European Program for Prevention necessary?

These results clearly indicate an urgent need for comprehensive programs focused on education (to promote lifestyle changes, invest in cardiovascular health as early as possible, participate in screening programs, receive vaccinations, and attend regular check-ups, etc.) and prevention. These initiatives aim to improve and reverse unfavorable trends by concentrating on how to avoid atherosclerosis progression and the diagnosis of atherosclerotic cardiovascular disease (ASCVD) and its complications, rather than solely addressing life-threatening complications such as myocardial infarction (MI), heart failure (HF), stroke, and severe PAD requiring intervention or amputation (11,12). To make these programs effective, it is essential to collaborate closely given the many similarities across countries in the CEE region—both in population risk factors and in healthcare systems, as well as deficiencies in health education, tradition, and culture (13). We should exchange ideas and experiences from existing screening and prevention programs, considering both their advantages and disadvantages (14,15). Based on these insights, there is a chance to create an optimal healthcare system focused on prevention that could be easily implemented not only in the CEE region but beyond. Additionally, there is an urgent need to disseminate and share knowledge about these programs, as insufficient communication has led to a lack of awareness of these often very successful healthcare solutions in Western countries. This need has paved the way for the introduction of the European Program for Prevention (EPP).

## Origin and Main Objectives of the EPP

The idea for the European Program for Prevention (EPP) (epprevention.eu) was proposed by Professor Maciej Banach and the members of the International Lipid Expert Panel (ILEP) (ilep.eu) in 2024. After extensive discussion, including its first presentation at the Three-Seas Congress in Lublin in March 2025, the EPP was officially established and launched during the ILEP Anniversary Congress in Warsaw at the beginning of June 2025. The main aims of the European Program for Prevention are:

- To enhance knowledge and awareness of validated best-practice preventive healthcare solutions already implemented in Central and Eastern European (CEE) countries, which face Europe’s highest burden of civilization-related diseases;
- To highlight the underestimated potential of the CEE region in advancing innovative preventive healthcare strategies;
- To evaluate whether existing successful healthcare solutions in the CEE region can be adapted and implemented in other countries (both within and beyond the CEE region) to establish a coordinated, comprehensive prevention framework;
- To demonstrate how program-recommended solutions could effectively reduce the burden of cardiovascular disease (CVD) risk factors, established CVD, and its complications, thereby minimizing reliance on costly therapies and procedures with limited clinical benefits;
- To shift healthcare priorities toward pre-disease prevention by reducing dependence on reactive, resource-intensive regenerative medicine and instead emphasizing early diagnosis, treatment, rehabilitation, and complication management.
- To link and integrate national registries that focus on at-risk individuals, and to develop new ones, using standardized variables and innovative AI-based data-collection methods, in order to build large, accessible epidemiological databases.
- To support existing programs—such as the EU Cardiovascular Health Plan (16) and the activities led by the World Heart Federation (WHF) (17) or World Health Organization (WHO) (18).

The latter aim is of special importance, as presenting the best examples of validated health solutions in cardiovascular prevention (along with their efficacy results) may help to better plan and implement strategies within the EU Cardiovascular Health Plan. With dedicated and appropriate financing, this could significantly impact the landscape of cardiovascular diseases in Europe. Additionally, it may support and complement the activities within the WHF or WHO initiatives for subsequent global implementation.

## The link between the EPP and other ongoing programs

The European Program for Prevention (EPP) can support the EU Cardiovascular Health Plan (as well as WHF/WHO strategic plans and activities) in several keyways, including:

1. *Promotion of Best Practices* (adoption of Evidence-Based Strategies)—the EPP promotes the implementation of successful preventive strategies and best practices derived from Central and Eastern European countries (19). These practices can be adapted and scaled across the EU to enhance cardiovascular health management.
2. *Early Detection and Risk Assessment* (ready-to-go screening protocols)—by emphasizing early diagnosis and assessment of cardiovascular risk factors, the EPP can contribute to the early identification and better (country-fitted) risk stratification of individuals at the cardiovascular risk. This aligns with the EU’s goal of reducing CVD incidence by enabling timely intervention and with the proposal to introduce an EU-wide Cardiovascular Health Check.
3. *Integrated Prevention Frameworks*—the EPP focuses on comprehensive prevention strategies that integrate lifestyle modifications, risk factor management, and multilevel customized educational programs. This holistic approach complements the EU Cardiovascular Health Plan’s aims to promote healthier lifestyles and reduce risk factors.
4. *Training and Education*—the EPP supports the training of healthcare professionals through initiatives like the EUPC, enhancing their understanding and capability to implement effective cardiovascular health interventions. Well-trained personnel can ensure that preventive measures are effectively communicated and executed.
5. *Cross-Border Effective Cooperation* (within shared resources and data)—the EPP encourages collaboration among EU member states (not only those from the CEE countries) for shared learning, enabling the exchange of data and successful intervention models related to cardiovascular health. This fosters a cooperative environment that can lead to improved health outcomes across borders with the CEE-country based examples to be implemented in whole Europe.
6. *Policy Development and Advocacy for Prevention Policies*—The EPP can help shape policies that prioritize cardiovascular prevention and CVD health by providing evidence and experience to support the creation and implementation of effective prevention policies within the EU framework.
7. *Monitoring and Evaluation* (within data collection and impact assessment)—with its focus on preventive measures, the EPP can aid in the systematic collection of data related to cardiovascular health interventions and outcomes (within the existing programs and outside), allowing the EU to evaluate the effectiveness of its Cardiovascular Health Plan and make necessary adjustments.
8. *Awareness Campaigns* – Public Health Initiatives—the EPP can support awareness campaigns aimed at educating the public about CVD risk factors and prevention strategies, fostering a culture of health consciousness throughout the EU. By aligning its objectives with the EU Cardiovascular Health Plan, the EPP can play a crucial role in streamlining and enhancing prevention efforts, ultimately contributing to a significant reduction in cardiovascular morbidity and mortality across Europe.

## How to use and implement the EPP?

Having established programs within healthcare systems, especially those that are already validated and equipped with quality indicators, it appears quite feasible to propose optimal CVD prevention programs for implementation in all countries. The primary limiting factors are financial resources (notably, primary prevention programs tend to be relatively inexpensive compared to those aimed at addressing existing diseases), the organization of the healthcare system (including whether changes are needed to ensure effective implementation), and the willingness of decision-makers to act. Unfortunately, many policymakers lack interest in long-term preventive programs, which typically require at least ten years to demonstrate significant population-level benefits.

**Figure 1:**
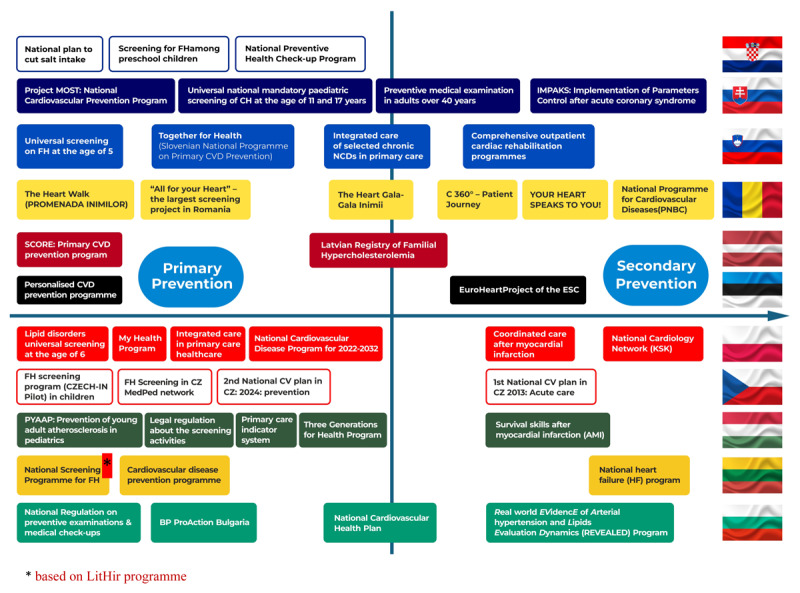
Examples of implemented preventive programmes in CEE countries.

Looking at the programs already available in Central and Eastern European (CEE) countries, we can easily propose an optimal system focused on CVD prevention, with measurable benefits for all non-communicable diseases. Such a system could include, for example, a mandatory Health Education subject introduced in primary and comprehensive schools in Poland (rather than being optional, as it is now). Additionally, a screening program for familial hypercholesterolemia in preschool children, successfully implemented e.g. in Slovenia and Croatia (20,21), in school children in Slovakia, and universal Lp(a) screening at age 18 as part of the National Cardiovascular Plan in the Czech Republic, should be included. This would also encompass a comprehensive check-up for all patients aged 20 and older, including Lp(a) testing, as introduced in Poland’s “My Health” Program. Furthermore, the system should integrate the Personalized CVD Prevention Program established in Estonia or the long-term validated “Together for Health” initiative (previously known as the Slovenian National Program on Primary CVD Prevention (22)), to ensure comprehensive and integrated care in primary prevention. The SCORE program for primary CVD prevention for individuals aged 40–65, which has been operating in Latvia since 2018, may also be referenced. In the event that these programs fail to prevent an atherosclerotic cardiovascular disease (ASCVD) event, patients should be swiftly enrolled in coordinated care following a myocardial infarction (KOS-Zawał), which has been effectively implemented in Poland for the past eight years (23,24), supplemented by the Hungarian educational program on survival skills after acute myocardial infarction (AMI). Subsequently, we must focus on effective programs for the comprehensive healthcare of patients with heart failure, which is now epidemic in many CEE countries. Notable examples include the National Heart Failure (HF) Program established in Lithuania and the National Cardiological Network (KSK) recently initiated after a pilot phase in Poland (25), as well as the full reimbursement of medical (including angiotensin receptor-neprilysin inhibitor (ARNI) and SGLT2-inhibitors), devices, interventional, and surgical treatment of HF in Romania. All these initiatives should be complemented by well-designed educational activities that engage prominent media figures and patient ambassadors, such as the “Hunting the Silent Killer” campaign in Croatia (70/26 campaign and “Do You Know Your Number?”), “The Heart Walk” and “The Heart Gala” in Romania. Furthermore, registries such as EuroHeart in Estonia (15), “All for Your Heart,” and SEPHAR in Romania (26), BP-ProAction and REVEALED in Bulgaria (27,28) should be utilized to continuously gather data, allowing for effective monitoring and opportunities for necessary improvements. This proposal is merely a starting point, considering the multitude of programs currently being implemented in CEE countries (29,30). Every healthcare system in Europe and beyond could benefit from these validated, ready-to-go solutions (Figure 1, Appendix Table 1).

## Final remarks

To conclude, considering the aforementioned epidemiological statistics on cardiovascular diseases, which clearly indicate a rising expectation of individuals presenting with risk factors and various types of CVDs, as well as complications such as acute coronary syndromes, strokes, PAD, arrhythmias, myocarditis, and HF, we must place substantial emphasis on prevention (31). This includes strategies to avoid disease and maintain heart and vascular health— such as utilizing the Simple Tips for the Healthy Heart (ILEP-SMILE) algorithm (32), implementing screening programs, and facilitating vaccinations to prevent numerous post-infection cardiac complications. Additionally, extensive health education is essential to improve therapy adherence (33), an independent risk factor for CVD. Enhancing communication among specialists is crucial to ensure that we treat patients holistically rather than merely addressing diseases. Such an approach, for instance, can help mitigate CVD events in cancer patients receiving innovative anticancer therapies (34). Therefore, we should learn from one another and utilize existing, validated, and improved programs that have demonstrated effectiveness in education, prevention, and therapy. There is no time for planning or revolution; resources are already available, and we need visionary and courageous decision makers to integrate them into a comprehensive healthcare system (avoiding commonly met siloing of existing programs) focused on prevention. Furthermore, we need politicians, experts, and the media to effectively communicate the risks associated with CVD, the leading cause of death globally. While there is fear surrounding cancer and infectious diseases, cardiovascular diseases are often underestimated (35). Through these programs and educational initiatives, with strong involvement from patient organizations and media, we must raise awareness through well-designed educational campaigns, teaching children and adolescents in schools to bring this knowledge home to help protect their parents and family members (36). We already have successful examples, and that is why the European Program for Prevention was launched.

## Additional File

The additional file for this article can be found as follows:

10.5334/gh.1491.s1 Appendix Table 1. Detailed description of existing preventive programmes in CEE countries, including their history, responsible entities, available results, and limitations.

## References

[1] Global Burden of Cardiovascular Diseases and Risks 2023 Collaborators. Global, regional, and national burden of cardiovascular diseases and risk factors in 204 countries and territories, 1990–2023. J Am Coll Cardiol. 2025;S0735-1097(25)07428-5. DOI: 10.1016/j.jacc.2025.08.015 40990886

[2] Koczkodaj P, Kuryłowicz A, Banach M, Bogdański P, Gałązka-Sobotka M, Kłoda K, et al. Uniting for the prevention and treatment of obesity – a call for coordinated, multisectoral action on a complex public health challenge. Arch Med Sci. 2025. DOI: 10.5114/aoms/211281 PMC12703476 41403622

[3] GBD 2021 Adult BMI Collaborators. Global, regional, and national prevalence of adult overweight and obesity, 1990–2021, with forecasts to 2050: A forecasting study for the Global Burden of Disease Study 2021. Lancet. 2025;405(10481):813–838. DOI: 10.1016/S0140-6736(25)00355-1 40049186 PMC11920007

[4] GBD 2021 Diabetes Collaborators. Global, regional, and national burden of diabetes from 1990 to 2021, with projections of prevalence to 2050: A systematic analysis for the Global Burden of Disease Study 2021. Lancet. 2023;402(10397):203–234. DOI: 10.1016/S0140-6736(23)01301-6 37356446 PMC10364581

[5] Banach M. No time to wait: Daily step counts should be incorporated into physical activity guidelines. Arch Med Sci. 2025. DOI: 10.5114/aoms/210673 PMC12703464 41403593

[6] Banach M, Lewek J, Surma S, Penson PE, Sahebkar A, Martin SS, et al. The association between daily step count and all-cause and cardiovascular mortality: A meta-analysis. Eur J Prev Cardiol. 2023;30(18):1975–1985. DOI: 10.1093/eurjpc/zwad229 37555441

[7] Banach M, Reiner Ž, Surma S, Bajraktari G, Bielecka-Dabrowa A, Bunc M, et al. International Lipid Expert Panel (ILEP). 2024 Recommendations on the optimal use of lipid-lowering therapy in established atherosclerotic cardiovascular disease and following acute coronary syndromes: A position paper of the International Lipid Expert Panel (ILEP). Drugs. 2024;84(12):1541–1577. DOI: 10.1007/s40265-024-02105-5 39497020 PMC11652584

[8] Gore R, Topp SM, Banach M, van Schayck OCP. What can we learn from developments in primary health care in south Asia? Lancet Glob Health. 2024;12(10):e1575–e1576. DOI: 10.1016/S2214-109X(24)00279-1 39178876

[9] Mensah GA, Fuster V, Murray CJL, Roth GA. Global Burden of Cardiovascular Diseases and Risks Collaborators. Global Burden of Cardiovascular Diseases and Risks, 1990–2022. J Am Coll Cardiol. 2023;82(25):2350–2473. DOI: 10.1016/j.jacc.2023.11.007 38092509 PMC7615984

[10] GBD 2023 Poland Collaborators. The burden of diseases, injuries, and risk factors by voivodship in Poland, 1990–2023: A systematic analysis for the Global Burden of Disease Study 2023. The Lancet Regional Health – Europe. 2025. DOI: 10.1016/j.lanepe.2025.101431 PMC12624802 41262419

[11] Banach M, Surma S, Guzik TJ, Penson PE, Blaha MJ, Pinto FJ, et al. Upfront lipid-lowering combination therapy in high cardiovascular risk patients: A route to effective atherosclerotic cardiovascular disease prevention. Cardiovasc Res. 2025;121(6):851–859. DOI: 10.1093/cvr/cvaf045 40098223

[12] Booth JN 3rd., Colantonio LD, Howard G, Safford MM, Banach M, Reynolds K, et al. Healthy lifestyle factors and incident heart disease and mortality in candidates for primary prevention with statin therapy. Int J Cardiol. 2016;207:196–202. DOI: 10.1016/j.ijcard.2016.01.001 26803243 PMC6311703

[13] Banach M. The International Lipid Expert Panel (ILEP)-the role of ‘optimal’ collaboration in the effective diagnosis and treatment of lipid disorders. Eur Heart J. 2021;42(37):3817–3820. DOI: 10.1093/eurheartj/ehab204 34079998

[14] Bedlington N, Abifadel M, Beger B, Bourbon M, Bueno H, Ceska R, et al. The time is now: Achieving FH paediatric screening across Europe – The Prague Declaration. GMS Health Innov Technol. 2022;16:Doc04. DOI: 10.3205/hta000136 36311985 PMC9583732

[15] Viigimaa M, Jürisson M, Pisarev H, Kalda R, Alavere H, Irs A, et al. Effectiveness and feasibility of cardiovascular disease personalized prevention on high polygenic risk score subjects: A randomized controlled pilot study. Eur Heart J Open. 2022;2(6):oeac079. DOI: 10.1093/ehjopen/oeac079 36600884 PMC9803971

[16] Fitzsimons D, Cosentino F, Linde C, Price S, Lüscher TF, Weidinger F. Delivering a European Union cardiovascular health plan: From building momentum to political action. Eur Heart J. 2025;ehaf164. DOI: 10.1093/eurheartj/ehaf164 40831371

[17] Laranjo L, Lanas F, Sun MC, Chen DA, Hynes L, Imran TF, et al. World Heart Federation Roadmap for Secondary Prevention of Cardiovascular Disease: 2023 Update. Glob Heart. 2024;19(1):8. DOI: 10.5334/gh.1278 38273995 PMC10809857

[18] Quiambao A, Malekpour MR, Golestani A, Heidari-Foroozan M, Ghamari SH, Abbasi-Kangevari M, et al. World Health Organization’s guidance for tracking non-communicable diseases towards sustainable development goals 3.4: An initiative for facility-based monitoring. EClinicalMedicine. 2025;85:103304. DOI: 10.1016/j.eclinm.2025.103304 40678696 PMC12269858

[19] Banach M, Mastalerz-Migas A, Wita K, Mysliwiec M. Poland takes a lead in effective lipid disorders management healthcare programmes in Europe. Am J Prev Cardiol. 2025 (in press). 10.1016/j.ajpc.2025.101346 PMC12663681 41321469

[20] Groselj U, Kovac J, Sustar U, Mlinaric M, Fras Z, Podkrajsek KT, et al. Universal screening for familial hypercholesterolemia in children: The Slovenian model and literature review. Atherosclerosis. 2018;277:383–391. DOI: 10.1016/j.atherosclerosis.2018.06.858 30270075

[21] Juričić G, Perović A, Matica J, Perković-Radojković K, Horvat V, Mandić D, et al. Analysis of total cholesterol results measured in the initial period of the Croatian screening program for familial hypercholesterolemia: A pilot study. Clin Chem Lab Med. 2025. DOI: 10.1515/cclm-2025-0536 40958753

[22] Vracko P, Maucec Zakotnik J, Govc Erzen J. Integrating evidence into practice: Examples from the Slovenia National Program on CVDs Prevention. Eur. J. Public Health. 2015;25(Issue suppl_3):ckv167.043. DOI: 10.1093/eurpub/ckv167.043

[23] Nowowiejska-Wiewióra A, Wita K, Mędrala Z, Tomkiewicz-Pająk L, Bujak K, et al. Dyslipidemia treatment and attainment of LDL-cholesterol treatment goals in patients participating in the Managed Care for Acute Myocardial Infarction Survivors program. Kardiol Pol. 2023;81(4):359–365. DOI: 10.33963/KP.a2023.0045 36871294

[24] Jankowski P, Topór-Mądry R, Gąsior M, Cegłowska U, Eysymontt Z, Gierlotka M, et al. Innovative managed care may be related to improved prognosis for acute myocardial infarction survivors. Circ Cardiovasc Qual Outcomes. 2021;14(8):e007800. DOI: 10.1161/CIRCOUTCOMES.120.007800 34380330

[25] Chioncel O, Čelutkienė J, Bělohlávek J, Kamzola G, Lainscak M, Merkely B, et al. Heart failure care in the Central and Eastern Europe and Baltic region: Status, barriers, and routes to improvement. ESC Heart Fail. 2024;11(4):1861–1874. DOI: 10.1002/ehf2.14687 38520086 PMC11287314

[26] Pop C, Fronea OFG, Pop L, Iosip A, Manea V, Dorobantu L, et al. High-normal blood pressure and related cardiovascular risk factors prevalence in the Romanian adult population: Insights from the SEPHAR III study. J Hum Hypertens. 2021;35(10):884–895. DOI: 10.1038/s41371-020-00417-z 32978496

[27] Postadzhiyan A, Yotov Y, Tisheva S, Nikolov F, Gruev I, Yaneva-Sirakova T. What potential cardiovascular risk reduction could be achieved with blood pressure and lipid lowering treatment? A simulation based on bp-proaction bg observational study. J. Hypertens. 2025;43(Suppl 1):p e233. DOI: 10.1097/01.hjh.0001117596.88971.43

[28] Postadzhiyan A, Yotov Y, Tisheva S, Nikolov F, Boychev D. Low-density lipoprotein cholesterol goal attainment in extreme cardiovascular risk patients – Frequency and relation with prognosis. Atherosclerosis. 2025;407(Suppl.):120163. DOI: 10.1016/j.atherosclerosis.2025.120163

[29] Laucevičius A, Rinkūnienė E, Petrulionienė Ž, Ryliškytė L, Jucevičienė A, Puronaitė R, et al. Trends in cardiovascular risk factor prevalence among Lithuanian middle-aged adults between 2009 and 2018. Atherosclerosis. 2020;299:9–14. DOI: 10.1016/j.atherosclerosis.2020.02.025 32179208

[30] Nagy GG, Mark L, Gerencser A, Reiber I, Kiss N, Rokszin G, Fabian I, et al. A nation-wide evaluation of suboptimal lipid-lowering treatment patterns among patients undergoing intervention for acute coronary syndrome in Hungary. J Clin Med. 2024;13(21):6562. DOI: 10.3390/jcm13216562 39518701 PMC11547159

[31] Banach M, Toth PP, Bielecka-Dąbrowa A, Lewek J. Primary and secondary cardiovascular prevention: Recent advances. Kardiol Pol. 2024;82(12):1200–1210. DOI: 10.33963/v.phj.103997 39775556

[32] Banach M, Fogacci F, Atanasov AG, Stoian AP, Jóźwiak J, Bytyçi I, et al. International Lipid Expert Panel (ILEP). A 360° perspective on cardiovascular prevention: The International Lipid Expert Panel SiMple tIps for the heaLthy hEart (ILEP-SMILE). Arch Med Sci. 2025 29;21(3):711–718. DOI: 10.5114/aoms/205732 40741254 PMC12305506

[33] Banach M, Penson PE. Adherence to statin therapy: It seems we know everything, yet we do nothing. Eur Heart J Open. 2022;2(6):oeac071. DOI: 10.1093/ehjopen/oeac071 36440352 PMC9683386

[34] Kuwabara M. The interplay between cancer and cardiovascular disease. Hypertens Res. 2025;48(3):1192–1194. DOI: 10.1038/s41440-024-02015-9 39543431

[35] Sobierajski T, Surma S, Banach M. Stroke and cancer – investigating the prevalence of fear among the polish population. Cross-sectional study. Eur Heart J. 2025;27(Issue Supplement_6). DOI: 10.1093/eurheartjsupp/suaf083.025

[36] Santos-Beneit G, Fernández-Jiménez R, de Cos-Gandoy A, Rodríguez C, Carral V, Bodega P, et al. Lessons learned from 10 years of preschool intervention for health promotion: JACC state-of-the-art review. J Am Coll Cardiol. 2022;79:283–298. DOI: 10.1016/j.jacc.2021.10.046 35057915

