# Supplementary material for: The European Program for Prevention (EPP) – Implementing Proven Preventing Measures Now!
Source: Glob Heart. 2025 Nov 14;20(1):103. doi: 10.5334/gh.1491 (PMC12617426; doi:10.5334/gh.1491)
Supplement: Appendix Table 1. — Detailed description of existing preventive programmes in CEE countries, including their history, responsible entities, available results, and limitations. [file gh-20-1-1491-s1.pdf]

COUNTRY	Name of the program	Description	Program launch date	Responsible entity	Available results and quality metrics	Limitations / comments
BULGARIA	**NATIONAL REGULATION on preventive examinations and medical check-ups**	Preventive examinations and tests organized according to the age of the population and lead by general practitioners who integrate the care and refer for specialized consultation. Based on a comprehensive interview individuals aged more than 18 years receives a set of diagnostic tests. Depending on the results, groups of individuals with an increased risk of developing specific diseases are formed and those individuals are further monitored and consulted by GPs and specialists. The groups are 1) increased risk for cardiovascular diseases, 2) increased risk for Diabetes, 3) Malignant neoplasm of the cervix, 4) Malignant neoplasm of the breast, 5) Malignant neoplasm of the rectosigmoid region and 6) Malignant neoplasm of the prostate For male 40+ and female 50+ in primary prevention settings, an additional test for the total, HDL, LDL and triglycerides (performed once every 5 years) together with a SCORE/SCORE2 evaluation are also available. Dispancerization (systematic health screening and preventive care process) for the patients with previous CV events with additional tests at yearly basis is led mainly by GPs	From 3.11.2016	National Health Fund / Ministry of Health (free for the patient)	Not available. According to NHIF data, in 2022, 2.3 million people underwent an annual preventive examination. A representative survey on the attitudes of Bulgarians towards annual preventive examinations, 32% of respondents said they last had an annual check-up this year, 31% said they did so last year, and 15% said they did so more than two years ago. 11% of respondents said they had never had an annual check-up.	The main limitation is the relatively small number of individuals taking advantage of the opportunities offered by preventive examinations. The idea of forming groups of individuals at increased risk of developing diseases, although a good one, in the absence of adequate funding does not fulfil its main goal of timely prevention. More focus on CVD prevention should be laid in the program, with comprehensive emphasis on the most prevalent CVD risk factors, like hypertension, lipid disorders, obesity, smoking or additional new factors like lipoprotein(a) and inflammation. The introduction of risk based strategies may influence the interest of practicing physicians.
	**National Cardiovascular Health Plan**	Main components 1) Primary prevention with a view to reducing mortality and morbidity 2) Secondary prevention through screening and early diagnosis 3) Early intervention, access to medical care and optimal treatment 4) Rehabilitation 5) Quality of life and other psychosocial outcomes across the spectrum of cardiovascular disease	12. 2024	Bulgarian Society of Cardiology and National Alliance for Cardiovascular Health	Not available. The specific plan is under construction.	A strong point is the leadership of main scientific organizations in the development of the national plan. The main idea is to fill the gaps in the existing algorithms in the country's healthcare system.
	**BP ProAction Bulgaria**	BP ProAction BG is a program of annual, comprehensive care intended for patients with difficult to control arterial hypertension. The program includes GPs, cardiologists and different specialists in the field of internal diseases and neurology. The main idea is to promote different techniques of out of office BP measurement and assessment of target organ damage together with risk-based strategies dedicated to better control of blood pressure and concomitant cardiovascular risk profile. The program includes educational activities for medical specialists and patients in order to achieve more deep involvement in the field of management of the disease. The main goal is to promote successful examples in the field.	2022 and ongoing	Bulgarian Hypertension League	Increase the number of controlled patients with difficult to control hypertension up to 70%. A comprehensive plan for stepwise approach based on TOD assessment and treatment provided to participating physicians	The plan includes to increase the number of GP involved in the program. Based on the results a set of additional laboratory and instrumental tests are proposed to the NHIF to be covered at national level
	**Real world Evidence of Arterial hypertension and Lipids Evaluation Dynamics (REVEALED) Program**	REVEALED is a program of annual, comprehensive specialist cardiology care in the scope of hospital and outpatient management (with the first control visit within 30 days) for patients after successful coronary revascularization. The first part of the program was the introduction of an electronic platform for lipids and BP values and treatment algorithms in order to promote the fastest achievement of guidelines directed goals for those extremely high risk patients. An algorithm for FH identification was conducted during the hospital stay. Together with the individual treatment plan, the second part includes extensive education on lifestyle, risk factors, cardiovascular diseases, and regular supervision of the additional investigations and treatment of the RF.	2024	Bulgarian Hypertension League	After the first year of introduction 30% of the hospitals preforming coronary interventions are included. The results clearly shows the unsatisfactory results of BP and lipid management in real practice	By linking the data with future CV events and procedures to change the practice of underutilization of guidelines directed therapies. The main perspective is to increase the number of participating centers.
CROATIA	**Zdravlje je bitno (National Preventive Health Check-up Program)**	National Preventive Health Check-up Program (starting November 11, 2025) Start of implementation: Monday, November 11, 2024, across Croatia Program implemented by: - General practitioners (family medicine doctors) - Health centres - Croatian Institute of Public Health (HZJZ) and county public health institutes Program goals: - Improve the health status of the population - Extend life expectancy and enhance quality of life - Increase health literacy - Reduce pressure on the healthcare system - Support long-term sustainability of the health system Key features: - Free of charge for all insured citizens, no additional cost - Check-ups performed by the chosen general practitioner - Based on: - Pilot project results conducted in 5 counties - Scientifically and professionally validated evidence	Started 11/2024 Across Croatia.	National Health Fund / Ministry of Health (free for the patient)	Not available for this programme but pilot project showed: Pilot project findings: Over two-thirds of examined citizens had health problems, including: - High blood pressure -Elevated blood sugar and cholesterol levels -Overweight -Harmful effects of smoking and alcohol One-third of people are ready to change unhealthy habits -Preventive care could reduce disease incidence by up to 40%	Economic impact: - Every 1 euro invested in prevention returns 14 euros to the healthcare budget
	**National plan to cut salt intake**	Started 2007. after results of national survey -- Epidemiology of hypertension in Croatia -- EHUH study- on national level with legislatives meat industry and bakeries reduced the amount of salt in their products	2007-2020	Croatian Hypertension society / Croatian atherosclerosis society	After 15 years of active promotion cutting salt intake results showed that on National, Croatian level, salt intake is reduced by almost 20%	Epidemiology of hypertension in Croatia 2 (EHUH2) finished in 2020. Showed this significant reduction of salt intake with clear repercussion on average blood pressure levels on population level. Less salt ingestion for sure potentiate this effect as well as better (but still modest improvement) in clinical inertia and usage of antihypertensive agents.
	**National programme: Screening for familial**	The target population are children patients aged 6 to 7 years as a part of obligatory national screening of preschool children Between 35 000-39 000 children screened annually Venous blood sample taken on an empty stomach analysing total blood count and Cholesterol (2023 and 2024 TC, 2025 LDL-K) The diagnosis of FH based on the Simon Broome Register Group. Patients with diagnosed	Pilot March 2023/ Started March 2024.	National Health Fund (the annual cost of the program is 3-4	Not available. Preliminary 10% children have Tv above 5.1mmol/l but lower than 6.1mmol/l About 1.4% of children are highly suspected on familial	Not all children do the test (despite it is obligatory); Participation rate is about 70-80%

COUNTRY	Name of the program	Description	Program launch date	Responsible entity	Available results and quality metrics	Limitations / comments
	**hypercholesterolemia among preschool children (Aged 6 and 7)**	hypercholesterolemia and positive family history for early cardiovascular events are referred to Pediatric care (secondary level) During the diagnostics of lipid disorders, it is important to exclude secondary dyslipidemia. Family screening is potentiated if child is positive on preschool screening (including family history of premature CV events)		million Euro)	hypercholesterolemia	
	**Hunting the silent killer [70/26 campaign and *do you know your number?]**	Prevalence of Hypertension and dyslipidemia is increasing in Croatia. In 2020 hypertension was killer number 4 and dyslipidemia killer number 7. In 2025. Hypertension [among women] is killer number 1 and dyslipidemia killer number 4 Prevalence of hypertension is 55% and dyslipidemia over 60% [LDL-C above 3 mmol/l].	Program started 2019.and from 2024 actively in media, networks involving ambassadors, public posters, painter busses, measuring blood pressure, cholesterol, glucose on public places...	Croatian hypertension league and Croatian atherosclerosis society	We examined over 20 000 people. They know their numbers! More to come.in future and in 2026 will repeat national survey to check the repercussion of performed measures on prevalence of hypertension, dyslipidemia. Does this way of education increase health literacy of nation.	
CZECH REPUBLIC	**1st National CV Plan**	Primarily focused on acute care.	2013		Not available.	
	**2nd National CV Plan**	Shifts focus to prevention strategies. Six strategic priorities guide this plan, including: - Availability of epidemiological data and quality indicators. - Primary cardiovascular prevention programs. - Ensuring availability and quality of care. - Highly specialized and centralized care systems. - Integration of cardiological care within the broader health system. - Support for research and scientific advancements in cardiology.	2024		Not available.	The National CV Plan 2024 requires involvement by the Ministry of Health of the Czech Republic, the Institute of Health Information and Statistics of the Czech Republic, the Czech Cardiological Society, healthcare payers, patient associations, and other governmental and non-governmental organizations.
	**National Cardiology Information System (NKIS)**	A key data-driven initiative for improving care. The National Cardiology Information System Portal is available online through the National Health Information Portal (https://www.nzip.cz/nkis). This system facilitates analyses, summaries, and access to critical data related to cardiovascular health.			Not available.	
	**General Preventive Examinations**	A general preventive examination is carried out every 2 years. The examination includes a comprehensive medical history, vaccination check, screening programs, physical examination, urine examination, ECG examination, and comprehensive laboratory examination. Specific laboratory examinations include lipid profiles, glycemia, liver tests, and ACR serum creatinine and glomerular filtration. The program also focuses on risk assessment, lifestyle recommendations, and disease management.			Preventive check-ups are well-covered in the child population (85-95%). However, there is a sharp decline in adults (40-55%).	Improving adherence to preventive examinations in the adult population.
	**FH Screening in CZ MedPed Network**	A network of 65 sites throughout the Czech Republic focused on screening for familial hypercholesterolemia (FH).			Not available.	
	**CzeCH-IN -- A Pilot Project of Universal FH Screening**	Newborn screening program involving LDL-cholesterol testing in umbilical cord blood followed by molecular genetic analysis if LDL-cholesterol levels exceed the 75th percentile.			Not available.	
	**CzeCH-IT - Check Cholesterol In Toddlers**	A pilot project for universal FH screening in preschool children (3-7 years) involving LDL-cholesterol testing in capillary blood.			Not available.	

COUNTRY	Name of the program	Description	Program launch date	Responsible entity	Available results and quality metrics	Limitations / comments
	**Universal Lp(a) Screening**	The National Cardiovascular Plan of the Czech Republic includes Lp(a) measurement into routine preventive check-ups. Screening will first occur at age 18, and at every first check-up with a GP if unknown. Rechecks should occur after menopause.			Not available.	
**ESTONIA**	**EuroHeart Project of the ESC**	Estonia was selected to contribute as the first pilot country in creating Europe's largest registry of cardiovascular diseases. The project will involve the development of a platform to help harmonise the collection of data related to cardiovascular diseases, increase the efficiency of analysing quality of care and support the execution of registry-based randomised cardiology trials. Lead by the Estonian Society of Cardiology, Estonia is taking part in the EuroHeart project initiated by the European Society of Cardiology. The project seeks to improve quality of care for patients with cardiovascular diseases. Cardiologists at Tartu University Hospital and North Estonia Medical Centre are participating in the EuroHeart project. EuroHeart annual report 2024 exposes details on patients with ST-elevation (STEMI) and non-ST-elevation myocardial infarction (NSTEMI) and their characteristics, treatments with percutaneous coronary interventions(PCI), medications and in-hospital outcomes.	By the end of 2024, fourteen countries have become members of the collaboration out of which eight participated in the registration of standardised data in 2023 which are included in this annual report.	European Society of Cardiology, Estonian Society of Cardiology,	The hospitals reporting to EuroHeart cover the whole Estonia. According to the mandatory Estonian myocardial infarction registry the quality differences in the indicators reported in EuroHeart would not be large, but some variation exists. Altogether, data from 63,961 patients admitted with myocardial infarction from the eight countries are included to the EuroHeart project.	National Registries of Estonia, France, Hungary, Iceland, Portugal, Romania, Singapore and Sweden are participating, and several more European countries will be included to the EuroHeart project.
**ESTONIA**	**Personalised CVD prevention programme**	From 2019 to 2023, the national project "Development of personalised cardiovascular medicine solutions in Estonia" has been carried out, Results have been published: Viigimaa M, Jürisson M, Pisarev H, Kalda R, et al. Effectiveness and feasibility of cardiovascular disease personalized prevention on high polygenic risk score subjects: a randomized controlled pilot study. Eur Heart J Open 2022; 2, 1--10. Necessary IT infrastructure was created to bring personalised medicine into common clinical practice in Estonia. While many proof-of-principle solutions, such as polygenic risk scores and extensive pharmacogenetic testing, have been effectively demonstrated in research projects, new IT components need to be developed and deployed to the national health system to integrate them into everyday clinical practice. Estonia has recently started the new study (2025-2030), which is the first of its kind globally, and its findings could enable people to receive personalised genetic risk-based treatment to maintain their heart health. The study is highly relevant worldwide, as cardiovascular diseases are the leading cause of mortality, according to the World Health Organization. We are going to establish preventive statin therapy as a standard practice for individuals with a high genetic risk of heart disease. The study will include 2,500 Estonian gene donors aged 45--80 who meet the genetic risk criteria for heart disease. The five-year study will be conducted by the University of Tartu, Tartu University Hospital, and North Estonia Medical Centre in collaboration with the Estonian Society of Cardiology, Estonian Society of Family Doctors, and pharmacies.	Starting 25 March 2025, the Estonian Biobank will invite selected gene donors to participate in an extensive heart health research study. The study aims to determine whether heart disease can be more effectively prevented with cholesterol-lowering treatment based on genetic risk assessment.	The project is supported by €15 million from the European Commission, with an equal investment from the Estonian state.	It is known that individuals with a high genetic risk are more likely to develop heart disease, and that they experience it earlier than those with a lower genetic risk. The difference is significant -- individuals with a high genetic risk have a 91% higher chance of developing heart disease, according to the Estonian Biobank data. This study will investigate whether preventive treatment based on genetic risk is more effective in preventing heart disease than current treatment practices.	Estonia is working to advance the implementation of cardiovascular precision medicine in Estonia by integrating clinical practice, genomics, IT, social sciences, and economics. Estonia is an excellent location for such studies, as a significant portion of our population are gene donors.
**HUNGARY**	**Primary care indicator system**	The General Practitioner (GP) Indicator System operated by the National Health Insurance Fund of Hungary (NEAK) plays a pivotal role in cardiovascular disease (CVD) prevention within the country's primary healthcare framework. This performance-based system incentivizes GPs to engage in preventive activities, early detection, and ongoing management of cardiovascular conditions. Cardiovascular Prevention Indicators in the NEAK System Several indicators within the NEAK system are directly related to cardiovascular health: Management of Hypertension: Regular monitoring and treatment of patients with high blood pressure. Lipid Profile Monitoring: Routine lipid testing for patients with diabetes mellitus and/or hypertension to assess and manage dyslipidemia. Care for Ischemic Heart Disease: Continuous follow-up and management of patients diagnosed with coronary artery disease. Diabetes Management: Monitoring of HbA1c levels and ophthalmologic examinations to prevent complications associated with diabetes. Prescription Monitoring: Tracking the prescription and redemption of lipid-lowering medications to ensure adherence to treatment protocols. These indicators are designed to promote proactive management of cardiovascular risk factors, aiming to reduce the incidence and severity of CVDs. Financial Incentives The NEAK Indicator System employs a performance-based financing model: Performance Scoring: GP practices are evaluated based on their adherence to the specified indicators, with scores reflecting the quality and effectiveness of care provided. Funding Allocation: Higher performance scores can lead to increased monthly funding for GP practices, thereby incentivizing the delivery of preventive and high-quality care. This model encourages GPs to prioritize preventive measures and chronic disease management, aligning financial incentives with public health goals. In summary, the NEAK Indicator System serves as a critical tool in Hungary's strategy to combat cardiovascular diseases, fostering a healthcare environment that emphasizes prevention, early detection, and effective management of cardiovascular risk factors.	The system was introduced in 2009, regularly updated, latest update July 2023.	National Health Insurance Fund of Hungary -- NEAK (free for the patient) https://www.neak.gov.hu/felso_menu/szakmai_oldalak/gyogyito_megeleozo_ellatas/alapellatas/indikatorrendszer-haziorvosi-fogorvosi	Improvement of prevention and care indicators: Since the introduction of the system, positive changes have been observed in adult and mixed practices, especially in the areas of diabetes and hypertension care, as well as lipid profile examinations. In addition, the proportion of women participating in mammography screening has also increased. Progress in pediatric practices: The number of anaemia and blood lipid screenings in pediatric practices has increased, as has the administration of meningococcal vaccinations. https://medicalonline.hu/haziorvostan/cikk/milyen_valtozasokat_hozhat_a_teljesitmenyertekeles_az_alapellatasban?	Data collection and feedback deficiencies: Filling out the patient records is often voluntary, which makes data collection and appropriate feedback difficult. In addition, deficiencies in the integration between medical software and informatic systems hinder the effective use of data. Funding inequalities between practices: During the introduction of the new system, significant differences were observed in the financing of practices, which necessitated compensatory measures. https://medicalonline.hu/haziorvostan/cikk/milyen_valtozasokat_hozhat_a_teljesitmenyertekeles_az_alapellatasban?
	**Legal regulation**	The Hungarian Ministerial Decree 51/1997 (XII. 18.) of the Ministry of Welfare establishes the	Hungarian	National Health Fund	Since the implementation of Ministerial Decree	There are still challenges, such as high rates of

COUNTRY	Name of the program	Description	Program launch date	Responsible entity	Available results and quality metrics	Limitations / comments
	**about the screening activities**	mandatory health screenings that general practitioners (GPs) are required to conduct within the framework of the national health insurance system. These screenings are designed to prevent diseases and detect them at an early stage, tailored to specific age groups. Among these mandatory screenings are the regular monitoring of cardiovascular risk factors, including blood pressure, blood glucose levels, and lipid profiles. These assessments are integral to evaluating an individual's cardiovascular risk and are essential components of preventive healthcare in Hungary. The decree outlines the specific screenings to be performed at various life stages, ensuring that individuals receive appropriate preventive care throughout their lives. For instance, adults are advised to undergo comprehensive health assessments at designated ages, which encompass evaluations of blood pressure, blood glucose, and lipid levels. These screenings are provided free of charge to insured individuals and are documented in the individual's health records. The decree also specifies the responsibilities of healthcare providers, including GPs, in conducting these screenings and ensuring that patients are informed about and receive the necessary preventive services.	Ministerial Decree 51/1997 (XII. 18.) of the Ministry of Welfare Since 18th December 1997	(free for patients)	51/1997, Hungary has made progress in certain areas of public health, notably in reducing infant mortality and increasing life expectancy.	cardiovascular disease and cancer, as well as regional health disparities, indicate the need for continued focus on preventive healthcare measures and equitable access to services.
	**Guideline for Preventive Consultations Conducted Within General Practice Collaboratives Professional Health Guideline of the Ministry of Interior on Preventive Consultations Targeting Adults Within General Practice Collaboratives Egészségügyi Közlöny**	Key Elements of Cardiovascular Prevention in the Guideline Assessment of Risk Factors: The guideline mandates the regular monitoring of cardiovascular risk factors---such as blood pressure, blood glucose, and lipid profile---among adults aged 40 to 65. Lifestyle Counselling: Within the framework of preventive consultations, general practitioners and members of the practice community provide lifestyle advice to patients, including support for smoking cessation, promotion of healthy eating, and encouragement of regular physical activity. Organization of Patient Support Groups: The guideline encourages the establishment of patient clubs aimed at educating patients and strengthening community support. Interdisciplinary Collaboration: Healthcare professionals working within practice communities, such as dietitians, physiotherapists, and psychologists, collaborate with general practitioners in implementing cardiovascular prevention strategies	31st March 2023	National Health Insurance Fund (free for the patient)	Since the introduction of structured preventive services in general practice communities in Hungary---particularly reinforced by the 2023 guideline---some initial and broader trends in population health have been observed, though long-term outcomes are still being monitored.	Key challenges in cardiovascular prevention within Hungarian primary care include inconsistent target achievement, regional disparities, limited use of allied professionals, poor patient adherence, and inadequate documentation and financial incentives. https://medicalonline.hu/haziorvostan/cikk/milyen_valtozasokat_hozhat_a_teljesitmenyertekeles_az_alapellatasban
	**Swiss--Hungarian Cooperation Program**	This program was implemented under the Swiss--Hungarian Cooperation Programme and aimed to prevent cardiovascular diseases among the Hungarian population. It included various screening examinations and health promotion activities, with a special focus on populations living in disadvantaged areas. The program contributed to the strengthening of primary care and the promotion of a preventive approach within the healthcare system.	2004-2012	Funded through the Swiss Contribution (Swiss--Hungarian Cooperation Programme), which supported projects in EU cohesion countries after 2004 EU enlargement.	- Improved access to cardiovascular screening (BP, lipid profile, glucose) in underserved and rural areas. - Strengthened primary care infrastructure and introduced prevention-oriented practices. - Promoted community-level health literacy and awareness of cardiovascular risk factors. However, results were mostly localized, without long-term national impact studies.	- Limited nationwide scalability: the program was project-based and time-bound. - Lack of long-term integration into the national health system. - Sustainability depended on local initiatives after funding ended. To enhance effectiveness: integrate into NEAK funding schemes, ensure continuous training for GPs, and embed community outreach into national prevention strategy. https://svajcialap.hu/en
	**Three Generations for Health Program**	Launched by Hungary's Ministry of Human Capacities, this nationwide initiative aimed to improve public health and prevent chronic diseases---particularly cardiovascular conditions---by integrating preventive services into primary care. Over 800 general practices organized into 143 practice communities participated, reaching nearly 1 million people. Key components included cardiovascular risk screening for adults aged 40--65, lifestyle counselling, and targeted health promotion for children and the elderly. The program strengthened interdisciplinary collaboration and contributed to the development of national prevention strategies through data collection and scientific publications.	2019-2021	Funded by the Ministry of Human Capacities (EMMI) and coordinated through National Health Insurance Fund of Hungary as part of the national primary care reform.	Reached nearly 1 million people through 143 practice communities; improved access to cardiovascular risk assessment (ages 40--65), lifestyle counselling, and preventive education; contributed to a more structured, community-oriented model of care; generated several scientific publications supporting prevention policy development.	Short program duration (2 years); lack of long-term funding and continuity; limited integration into permanent national frameworks; for greater impact, prevention activities should be institutionalized, financed long-term, and extended to wider age groups with sustained interdisciplinary support. https://www.gokvi.hu/harom-generacioval-az-egeszsegert-program-kardiovaszkularis-prevencio-az-alapellatasban
	**CÉL1 programme**	We have sought to promote the effectiveness of cardiovascular disease prevention by organising the Hungarian Cardiovascular Consensus Conference and by widely disseminating the recommendations of the Consensus Conference. The first such Consensus Conference was organised in 2003 by 9 scientific societies, 13 in 2005, 15 in 2007 and the most recent VIIIth Hungarian Cardiovascular Consensus Conference 2020. In November 2005, 19 scientific societies participated and agreed on recommendations that, taking into account the latest evidence and therapeutic options, defined the target values for blood pressure, lipids, blood glucose, BMI, abdominal circumference for each cardiovascular risk category and detailed lifestyle and therapeutic recommendations, which were	Cardiovascular Consensus Conference started from 2003, the last VIIIth Hungarian Cardiovascular Consensus Conference was	Supported by the Federation of Hungarian Medical Societies and Associations (MOTESZ)	The consensus allowed for uniform communication among cardiovascular prevention companies. All companies advertised the same target values in the given cardiovascular risk category.	Only small proportion of patients took part in the program.

COUNTRY	Name of the program	Description	Program launch date	Responsible entity	Available results and quality metrics	Limitations / comments
		widely disseminated to ensure that new effective therapies are incorporated into everyday practice as soon as possible. (Gy Paragh, I Karádi, Metabolizmus XIX., 2021,pp: 27--33.)	2020			
	**CÉL2 programme**	We looked at the effectiveness of the implementation of these consensus conference recommendations in practice in the framework of the CÉL Programme.			We widely published the consensus recommendations and those evidence that supported the recommendations nationwide.	
	**CÉL3 programme**	18 142 patients of 300 GPs in the CÉL1 programme, 24 000 patients of the 120 GPs in the CÉL2 programme, and 11 824 patients of the 68 GPs in the CÉL3 programme were analysed. The cholesterol target attainment rate in patients at high cardiovascular risk increased from 11.5% to 22.7% in 2005 compared to baseline in 2004, and to 30.1% in 2006. The BMI target was reached by 22% of patients, the blood glucose target by 17.8% and the blood pressure target by 54%. (Gy. Pados, I. Karádi, Gy Paragh: Metabolizmus 2008;VI., 4: 208-213.)			Tens of thousands of individuals have participated in the programs, resulting in an improvement in the proportion of patients achieving their target values. The effectiveness of lipid-lowering treatment applied by doctors has improved.	
	**REALITY Study**	In the REALITY (The Return on Expenditure Achieved for Lipid Therapy) Study, we investigated the daily practice of GPs and specialists in lipid-lowering treatment. We analysed data from around 440 patients of 30 GPs and 14 cardiologists and/or lipidologists. 74% of the patients received lipid-lowering treatment and only a small percentage achieved the desired target. A higher percentage of patients treated by specialists achieved the therapeutic target. (Gy Paragh, L Márk, K Zmolyi, Gy Pados, P Ofner: Drug Invest 2007;27(9)647-660.)			Factors influencing patient compliance can play an important role in achieving target values and their regular consideration is necessary for more effective therapy.	
	**MULTI-GAP (MULTI Goal Attainment Problem) Study**	In the MULTI-GAP (MULTI Goal Attainment Problem) study, data from 2332 patients with cardiovascular events were processed. Serum total cholesterol, LDL, HDL, triglyceride levels were assessed, and it was found that 15% of the patients studied did not receive lipid-lowering treatment, 44% of patients treated by specialists achieved the LDL target, 61% of patients at high risk achieved the HDL target, while 43% of patients achieved the triglyceride target. The results suggested more effective lipid-lowering treatment, higher-dose statin use, and combination drug therapy. (I Reiber, Gy Paragh, L Márk, Gy Pados: Orvosi Hetilap 2011;152, 21: 822-827.) In 2011 and 2012, MULTI-GAP examined patient medication compliance with physician prescribing habits. It found that medication cooperation and goal achievement showed a linear relationship. Based on an analysis of data from around 450 000 patients, statin persistence showed that 54% of patients stopped therapy in the first month, 38% remained on statin therapy after the second month, only 27% after six months and 20% after one year. Among patients with acute coronary syndrome, 76% of patients were still on statins at 1 month, 62% at 6 months and 50% at 1 year. (L Márk, I Reiber, Gy Paragh, I Karádi, Gy Pados, Z Kiss: Metabolizmus XI., 3. 2013:177-181) The MULTI-GAP 2013 study investigated the adherence and persistence of statin therapy in patients with atherosclerotic disease. Data from 1519 patients were processed for the study. The study showed that the true persistence of patients assessed by their treating physicians as having 100% persistence was 74%, 25% lower than the true persistence. And the adherence rate was 36%. (G Simonyi Orvosi Hetilap 155(17) 2014:669-675.) We investigated the relationship between daily practice and recommendation in 12 997 patients with acute coronary syndrome undergoing revascularisation. Our study showed that lipid-lowering treatment was reduced during follow-up. Higher-intensity lipid-lowering treatment had higher persistence and adherence and better mortality. Lower statin dose use was observed more frequently for several diseases. (G Gy Nagy, L Mark, A Gerencser, I Reiber, N Kiss, Gy Rokszin, I Fabian, Z Csanádi, I Karádi, D Aradi, L Bajnok, Gy Paragh: Journal Clinical Medicine 2024;13, 6562.)			Not available.	
	**Survival skills after myocardial infarction (AMI)**	The programme is a structured training for patients and complex secondary prevention behavioural approach for patients with acute myocardial infarction treated at PCI centres throughout Hungary. Video presentations are held for each patient willing to participate in the program and they receive electronic and / or hard copies of training materials. A trained education facilitates the training and if necessary, follows the patient.	It was introducing in 2018 and ongoing currently	Hungarian Society of Cardiology	The Hungarian Myocardial Infarction Registry (HUMIR) was updated to include input from all PCI centres whether the treated patients participated in structured training programs.	The structured education at an acute care setting requires substantial resources, therefore not all PCI centres participate. Based on HUMIR about 25% of AMI patients were enrolled into the program and data was missing from 30%. COVID had a significant negative impact on the program.
	**Lipid disorders**	In 2023, the Hungarian Atherosclerosis Society Children's Section founded PYAAP (Prevention of young adult atherosclerosis in paediatrics), which aims to identify children and adolescents at risk of atherosclerosis, to reduce the risk factors, in order to prevent cardiovascular disease. To this end, national and regional centres have been set up. With regional centres in Northern Hungary, South-West Hungary, West Hungary, East Hungary and South-East Hungary. In the case of a higher cholesterol level detected by a paediatrician, school doctor or general practitioner, the child or adult is	2023		It promotes the screening and treatment of early cardiovascular risk factors. Early screening and treatment of FH patients thus reduces cardiovascular complications. The state supports the treatment of very high and high cardiovascular patients with the latest preparations. The	Improving the engagement of healthcare personnel involved in screening.

COUNTRY	Name of the program	Description	Program launch date	Responsible entity	Available results and quality metrics	Limitations / comments
		referred to the regional lipid centre, which is run by colleagues with lipidology, obesity, hypertension and diabetes licensing exams. At these centres, the cause of the lipid abnormality is precisely defined, and, on this basis, therapy is started, with detailed lifestyle advice. The colleagues working in the centers have a lipidology license exam. Registration of patients with familial hypercholesterolaemia. The Dutch criterion is used to define familial hypercholesterolaemia. Statin is the first-line therapy for cholesterol reduction. If the target value is not reached with statin treatment at the maximum tolerated dose statin + ezetimibe combination recommended. If the statin + ezetimibe combination is not sufficient PCSK9 inhibitor treatment add to the previous lipid lowering therapy. These therapies are available to patients with state subsidies. Among the lipid-lowering drugs, ezetimibe is indicated for patients with cardiovascular disease (ACS, STEMI, non-STEMI), patients with a proven history of cerebrovascular events (TIA, stroke), patients undergoing surgery for peripheral vascular disease, patients at very high cardiovascular risk and with hyperlipidaemia who have received at least 20 mg rosuvastatin or at least 40 mg atorvastatin for 3 months and who have not achieved target LDL levels or have proven intolerance to statin. PCSK9: In a supported indication, it may be prescribed by designated academic centres for the treatment of HoFH or for secondary prevention in patients with LDL levels above 1.8 mmol/l despite 3 months of maximum tolerated dose statin therapy and 1 month of ezetimibe therapy, or in combination with statin and other lipid-lowering therapy. May be confirmed in case of statin intolerance. Bempedoic acid is currently not available in our country. Inclisiran in a supported indication, designated university centers can prescribe for the treatment of HoFH, or for the treatment of secondary prevention patients whose LDL level exceeds of 1.8 mmol/l, despite 3 months of maximum tolerated dose statin treatment and 1 month of ezetimibe treatment with a statin, or a statin and other lipid-lowering therapy combined. Can be proven in case of statin intolerance.			government supports the lipid lowering therapy of patients with very high and high cardiovascular risk. The reimbursement of the new expensive lipid lowering therapy by the government.	
LATVIA	**SCORE**	Primary CVD prevention programme (ages 40–65). Launched in 2018, the programme uses proactive screening (symptoms, family history, smoking status, blood pressure, BMI, cardiac auscultation, and blood lipids and glucose), followed by cardiovascular risk calculation using SCORE and subsequent lifestyle advice, diagnostic testing, and drug treatment where indicated. The State Methodological Guidance Authority in Cardiology initiated an update to the programme in late 2025 following publication of the 2025 Focused Update of the 2019 ESC/EAS Guidelines for the Management of Dyslipidaemias.	Since 2018	Ministry of Health (National Health Service)	Not available.	When SCORE-2 was introduced, most individuals aged 40–65 in Latvia were classified as high or very high risk, which created uncertainty regarding the usefulness of risk calculation and contributed to a low response rate. The updated 2025 definitions of high and very high risk according to SCORE-2 will enable more accurate stratification of individuals and are therefore expected to be adopted more widely by general practitioners. In addition, risk enhancers are planned to be incorporated into future decision algorithms, and bureaucratic obstacles will be minimized.
	**Latvian Registry of Familial Hypercholesterolemia**	The Latvian Registry of Familial Hypercholesterolaemia (FH) is a nationwide registry established in 2015. Referrals are coordinated via a central coordinator phone line, providing access to consultations with cardiologists specialised in lipidology. Since 2025, pilot programmes of universal and opportunistic screening have been conducted within the framework of the Joint Action on CARdiovascular diseases and DIabetes (JACARDI). The registry also captures other primary dyslipidaemias—notably elevated lipoprotein(a) [Lp(a)] and severe hypertriglyceridemia - and collaborates with the Genome Database of the Latvian Population (LGDB) and paediatric endocrinologists, with ongoing international engagement.	Since 2015	Mostly research grants.	More than 1500 patients in database. Estimated 10% FH cases detected.	There is currently no state-funded national programme, and reimbursement for genetic testing is limited; a dedicated paediatric FH registry is needed.
LITHUANIA	**Cardiovascular disease prevention programme**	The frequency of the patient's participation in the programme depend on the risks identified. If the risk of a cardiovascular disease is found to be low or moderate, the person will be invited to take part in the programme again after 4 years. If the risk of these diseases is high, the next time they will be invited after 2 years, and if the risk is very high, after 1 year. If a very high risk is identified, a referral will be made by a family doctor to a cardiologist for a further comprehensive examination of the likelihood of a cardiovascular disease. If a family doctor determines that the patient is at high or very high risk of a cardiovascular disease, the patient will be given a primary prevention plan. This will include targeted patient's body mass index, arterial blood pressure and low-density lipoprotein cholesterol concentration in blood, ways and terms for their control, and recommendations for lifestyle changes. After 6 months, the patient will have to revisit his/her family doctor, who will assess the results of this plan and adjust the plan.	December 2005.	National Health Fund / Ministry of Health (free for the patient)	Hygiene Institute () shows that the number of people who develop and die from cardiovascular diseases in our country is increasing every year. Almost 914 thousand of Lithuanian residents developed these diseases in 2021 and more than 931 thousand -- last year. Last year alone, 23 000 patients died of cardiovascular diseases. Therefore, prevention of these diseases turns to be very important.	At the moment, participation in the cardiovascular prevention programme is not sufficient. According to the data of the National Health Insurance Fund under the Ministry of Health (NHIF), less than half of targeted Lithuanian residents (46 pct.) participate in this programme annually. Earlier, men aged 40-55 and women aged 50-65 could participate in the cardiovascular prevention programme. Age limits for target population have now been extended and men and women aged between 40 and 60 (inclusive) may participate in the programme.
	**National Screening Programme for Familial hypercholesterola**	The Lithuanian national FH screening programme was implemented in 2016 and was created mainly on the basis of the Lithuanian High Cardiovascular Risk (LitHir) primary prevention programme. Opportunistic screening approach guided by increased levels of LDL-C has been chosen as the main method to detect possible index cases in general population. An electronic extraction tool was applied to the database of lipidograms of the LitHiR programme, then statistical analysis of	2016-	National Health Fund	According to LitHir analysis of the period 2009 - 2016, 1 in 67 middle-aged patients have likely FH phenotype in Lithuania. Updated period (2009 - 2022) shows that the prevalence FH-like phenotype in middle-aged Lithuanian population is	Although FH screening programme (based on LitHir programme) provides the opportunity to screen a large portion of the Lithuanian middle-aged population, it is also notable that such participation is still not active enough. Additionally, due to attachment to LitHir, in many

COUNTRY	Name of the program	Description	Program launch date	Responsible entity	Available results and quality metrics	Limitations / comments
	**emia (FH)**	retrospective/cross-sectional data has been performed. During the period of 2009 - 2022, 234,779 lipid profiles of middle-aged individuals in Lithuania were analyzed to identify cases of phenotypically probable familial hypercholesterolemia (FH). Since 2018, selected patients, who signed informed consent forms, have been included in the Lithuanian long-term FH observation programme, which is a part of the EAS-FHSC global registry. The coordinating centre of the programme was set to be the tertiary care hospital with specialized cardiovascular prevention unit - Vilnius university hospital Santaros klinikos; however, patients with FH are referred here for cardiology consultations from across Lithuania. In 2020 Children's Hospital (affiliate of Vilnius University Hospital Santaros Klinikos) has become a part of the programme.			approximately 1:86. In 2021 Centre of rare diseases of Lithuania has received a grant for 1000 children (from 5 to 10 years) to be screened for FH. The first results are already published (https://pubmed.ncbi.nlm.nih.gov/40282906/).<br><br>When analysing the data of FH long-term observation programme, the median age at FH diagnosis was 47 years, and 13% of the included patients were diagnosed with coronary artery disease, which is nearly twice the prevalence of CAD in the general Lithuanian population, which was 6.97% in 2022, as estimated based on data available from the Lithuanian Health Education and Disease Prevention Centre.	cases, this screening model relies heavily on patients' own interest in their health since participation in LitHir is not obligatory. For most patients with FH, dyslipidemia is "silent" and does not cause any symptoms, which may result in some patients being reluctant to adhere to treatment or start treatment altogether. Unfortunately, despite all the efforts, the availability of genetic testing in Lithuania is still limited, as only 30% of patients (who were included in FH long-term observation programme) were able to be tested for FH-causing mutations. Such barriers to screening should not be overlooked and should be addressed in the future.
	**National heart failure (HF) program**	The purpose of training HF patients is to provide them and their family members with special knowledge about HF disease, its course and care methods, and recommendations for the formation of lifestyle and self-care skills. Consultations are provided based on a referral from a cardiologist, family doctor, or internal medicine doctor. A patient with HF may have access to up to four consultations within a 12-month period. It's advisable for patients to receive the first consultation within 30 days after being discharged from hospital or upon receiving a referral for these consultations from their doctor. Consultations are provided only after active treatment of a chronic disease has been provided, in the following indications: - patients after treatment in hospital for exacerbation of HF, patients with HF of functional class I--IV (according to the New York Heart Association (NYHA) functional capacity class), who have been diagnosed with primary or concomitant diseases, designated by codes I50, I11.0, I13.0, except for patients whose diagnosed underlying disease is designated by ICD-10-AM codes I21--I22 or I60--I69 and who were provided with inpatient care and supportive treatment during one calendar year. - patients after the provision of active chronic disease treatment by a cardiologist due to the first-time diagnosis of heart failure (I50) due to established cardiovascular diseases, designated according to ICD-10-AM codes I20.1, I20.0, I25.5, I11.0, I13.0, I13.2, I42, or when a heart transplant is indicated for the patient, except for patients who inpatient care and supportive treatment were provided during a period of one calendar year.	26th October 2015	National Health Fund / Ministry of Health (free for the patient)	In Lithuania, it is estimated that over 120,000 people have HF. 1-year data: -incidence of HF (number of newly diagnosed patients) -- 15.077; -incidence of HF (number of patients) -- 86,817; -incidence of hypertensive heart disease with heart failure (congestive) (number of patients) -- 101.334; -15.5% of patients with HF die within one year; -14.1% of patients with HF are treated in hospital more than once during the year.	Not all patients complete the training program.
**POLAND**	**Health Education subject**	Health Education has been introduced as a new subject in all Polish primary and comprehensive schools, replacing family life education. Classes are held in years 4-8 of primary schools and years 1-3 of secondary schools (high schools, technical schools, and vocational schools). Although originally intended to be mandatory, health education will unfortunately be optional (we predict that less than 20% of pupils will finally attend) in 2025/2026 schoolyear. The aim of the course is to develop students' competencies related to lifelong health maintenance and to build individual and community health potential, including detailed knowledge on the diagnosis and understanding of lipid disorders and atherosclerosis as a cause of atherosclerotic cardiovascular disease (ASCVD), on how to monitor lipid profiles, how to prevent with effective lifestyle changes (regular exercise, diet, preventing underweight and obesity) and why therapy adherence is of critical importance for effective treatment.	1st September 2025	Ministry of Education	The initial data suggest that due to the lack of obligation, this subject has been chosen by only about 37% of all pupils (by Sept. 30th).	Lack of mandatory. Limited number of pupils attend the subject.
	**My Health (previously Prevention 40+)**	Based on a comprehensive interview and implemented in primary healthcare facilities. Patients aged 20-49 years have preventive balance tests performed once every 5 years, while in the case of patients >49, they are performed once every 3 years. The basis for the tests is to be a questionnaire, and based on the answers, the patient receives a set of diagnostic tests. After the tests, the patient meets with the medical personnel and discusses the results. They receive recommendations regarding: a healthy lifestyle, diet and physical activity, preventive examinations, individual recommendations resulting from identified risk factors, a schedule of recommended vaccinations, other recommended health interventions (Individual Health Plan). All test results are reported to the individual patient account (IKP application) and - with the patient's consent - other physicians have access to them. For people 60+, an additional test for the diagnosis of dementia are also available. Available laboratory tests in the program: basic and extended scope, including blood count, glucose, creatinine, lipid profile, TSH, liver enzymes, total PSA, anti-HCV, lipoprotein(a).	This comprehensive preventive programme replaced the programme 'Prevention 40+' (that was introduced in June 2021) since 5th May 2025.	National Health Fund / Ministry of Health (free for the patient)	Not available. By Nov. 2024 - 4.77 million people received an e-referral and 3.9 million were tested as part of the program, including 2.6 million patients up to 65 years of age (20% of this age group), 1.3 million patients ≥65 years of age (17% of people in this age group), 759 thousand people were re-tested.<br><br>Till mid-October 2025, 1.5 million Poles have filled out the questionnaires under the "My Health" program. More than half of the program participants are women aged 20–59, while men over 60 are the least likely to use the screenings	The previous version of the program -- Prevention 40+ was limited as a patient was left with the test results without any further consultation; moreover, only small proportion of patients took part in the program finally (about 20%) as a result of lack of suitable education campaigns. 'My Health' seems to learn from the limitations of the previous version but again if one would like to have this program successful, we need to extensively educate to encourage people, as early as at the age of 20+ to check and invest in their health.
	**Integrated care in primary care**	Within the program primary healthcare physicians have been given the opportunity to refer patients for specific comprehensive, as well as to educational advice (provided by a nurse or doctor), dietary	1st October 2022	National Health Fund	According to data from the National Health Fund (April 2025), approximately 40% of primary health	They are still 60% primary care clinics that do not participate in the program. Some of them have difficulties

COUNTRY	Name of the program	Description	Program launch date	Responsible entity	Available results and quality metrics	Limitations / comments
	**healthcare**	advice and specialist consultations as part of coordinated care. The main goals of coordinated care are systematizing patient care, expanding the diagnostic capabilities of primary healthcare; shortening the patient's path to obtaining appropriate services and the possibility of consultations within certain specializations. Also individualized treatment planning, intensifying the implementation of preventive programs, including by actively encouraging patients to report for tests, providing feedback to the ordering physician through closer cooperation with specialists, improving the quality of patient care through health education, and increasing dietary support for patients. Coordinated care services are provided within five paths: cardiology, diabetology, pulmonology/allergy, endocrine, nephrology. To implement the selected coordinated care path, the primary care facility must provide the possibility of consultation with a specialist in a given field, which takes place in two formulas: a stationary visit or a remote consultation. It is also necessary to provide access to dietary consultations. A primary care physician is required to conduct an annual comprehensive visit, which is the basis for assessing the patient's current condition and developing an Individual Medical Care Plan. This provides the patient with developed guidelines for pharmacological and non-pharmacological treatment, as well as planned tests, consultations and educational advice. The coordinator in each centre is responsible for the entire diagnostic and therapeutic process of the patient.			care clinics have already concluded agreements to provide coordinated care. In November 2024 'My Patients' Foundation presented the results of the survey on the program: as many as 80.2% of respondents would recommend participation in a coordinated care program to their relatives or friends; >93% of patients confirm that diagnostics and treatment were going according to plan -- importantly primary health care facilities within the program provide access to diagnostic tests in several times shorter time than in the queue for them in outpatient specialist care.	to provide specialists consultation and educational advises. More focus on CVD prevention should be laid in the program, with comprehensive emphasis on the most prevalent CVD risk factors, like lipid disorders, obesity, smoking or lipoprotein(a).
	**Coordinated care after myocardial infarction (KOS-Zawał)**	KOS-Zawał is a program of annual, comprehensive specialist cardiology care in the scope of hospital treatment, outpatient treatment (with the first control visit within 14 days) and rehabilitation intended for patients after myocardial infarction. The coordination of the patient's treatment process is handled by a dedicated Coordinator, who supports the patient throughout the program. The program consists of three stages: hospitalization including diagnostics and treatment of the MI acute phase, cardiac rehabilitation, inpatient or outpatient (selected by a cardiologist), and outpatient cardiology care, including cardiology consultations (4 visits) and detailed diagnostic tests (including echocardiography). It ends with summary advice including laboratory tests and a summary of the clinical condition. Patients receive an individual treatment plan, extensive education on lifestyle, risk factors, CV diseases, and supervision of lipid disorder treatment.	1st October 2017	National Health Fund (68,000 patients benefited by end 2022, total cost PLN 1.25 billion ~300 million Euro)	By July 2024, 112 contracts signed; >30% MI patients covered by the program. Cardiac rehabilitation started during or within 60 days post hospitalization reduced death risk by 57%. One cardiology consult reduced death risk by 30%, two or more by 37%.	Still 70% MI patients not covered (facultative program); program lasts 12 months but should extend to 24-36 months based on patient risk.
	**National Cardiology Network (KSK)**	The structure of the KSK (dedicated for patients in CVD secondary prevention) will consist of: (1) cardiology centers of the 1st level of providing cardiological care (OK I), (2) level II cardiology centers providing cardiological care (OK II) and (3) cardiology centers of the 3rd level of providing cardiological care (OK III). OK I, within the KSK, will act as centers providing mainly basic cardiological diagnostics and treatment. Both specialist clinics and multi-profile hospitals will be qualified to the OK I level. Centers classified as OK II in the KSK will ensure coordination and continuity of cardiological care, especially in the field of comprehensive cardiological diagnostics (cardiology clinic) and cardiological treatment (cardiology department). They will also provide or provide access to the hemodynamics laboratory or interventional radiology laboratory, as well as to services in the cardiac rehabilitation department and in the day-care facility, center or department of cardiac rehabilitation. OK III are centers qualified for the highest level of cardiological care - they are intended to ensure coordination and continuity of cardiological care, in the field of comprehensive cardiological diagnostics (cardiology clinic), cardiological treatment (cardiology department) and cardiac surgery treatment (cardiac surgery department and anaesthesiology and intensive care department). OK III will also provide multidisciplinary cardiological treatment, including in the department of internal diseases or vascular surgery or neurology, as well as an access to health care services in the hemodynamics laboratory or interventional radiology laboratory, electrophysiology laboratory, cardiac rehabilitation department and in a day-care facility, center or department of cardiac rehabilitation. Patients' e-KOK card within the program is intended to help properly coordinate the treatment process, monitor the quality of cardiac care and ensure the proper flow of information regarding the implementation of cardiac diagnostics and treatment.	The National Cardiology Network was initiated by the act of June 4, 2025, which was announced on June 17, 2025; however, the process of creating the network began earlier with a pilot program that took place from 2021 to 2024.	Ministry of Health/National Health Fund	Not available.	
	**Lipid disorders universal screening at the age of 6**	The target population are children patients aged 5 to 7 years, reporting for a balance examination at annual compulsory preschool preparation. Tests are based on venous blood after fasting with parental consent. Diagnosed based on Simon Broome Register Group criteria. Positive pediatric patients referred to lipid clinics and cardiology centers. Important to exclude secondary dyslipidemia.	5th May 2025	National Health Fund (annual cost 3-4 million Euro)	Not available. Expected to identify 17.5-27k suspected FH, about 1,400 new FH diagnoses annually in children.<br>Based on October 2025 estimated data, about 3,000 children from the program have already been referred to Children's Lipid Clinics.	About 25% children do not participate in the 6-year old's examination despite obligation; limited, not fully reimbursed access to genetic tests and consultations, long waiting times.
**ROMANIA**	**National Strategy for Management of Cardiovascular**	National strategic framework and action plan for improving care and prevention of cardiovascular diseases and stroke.	2025-2030	Romanian Government	Main objective is to decrease cardiovascular mortality by 30% in the next 5 years. Main actions in the prevention area include initiation of at least	Success depends on intersectoral collaboration and sustained funding. Implementation across diverse healthcare settings; monitoring progress towards 2030

COUNTRY	Name of the program	Description	Program launch date	Responsible entity	Available results and quality metrics	Limitations / comments
	**and Cerebrovascular Diseases**				20 regional prevention centres (covering half of the counties); increase of the early diagnosis of CCVD; development of online platforms for patients. Meanwhile, it aims to expand access to care in underserved regions and to reduce time from symptoms-to-intervention by at least 20%. It includes a strategic framework and a detailed funded plan of approximately 200 million Euro.	targets.
	**National Programmes for Cardiovascular Diseases**	A national program offering curative services in cardiovascular and cerebrovascular diseases, including interventions and surgery.	2004-ongoing	Ministry of Health, National Health Insurance House	Provides interventional cardiology and neurology, electrophysiology, and cardiac surgery services. Includes dedicated programs for acute coronary syndromes and stroke. Plans for at least 50% budget increase between 2025-2030, periodic updates to eligibility criteria and treatment protocols, and expansion of specialized units in underserved areas. The program contributed to reducing in-hospital mortality in the acute coronary syndromes and stroke.	Uneven distribution of services. The need to expand access to services in underserved areas and periodically update protocols.
	**"All for Your Heart" National Cardiovascular Risk Screening Programme**	Targeted screening for cardiovascular risk factors among vulnerable populations.	2020-2023	EU project of 20 million Euro	165,001 people benefited from screening, of which 85,000 from rural areas. Cardiovascular risk distribution (SCORE) was: 22% low risk, 58% moderate risk, 15% high risk, and 3% very high risk. Prevalence of risk factors in the tested population was: 55% with elevated total cholesterol, 15% with elevated fasting blood glucose, 76% overweight or obese (36% obese), 17% smokers, 40% physically inactive, and 26% alcohol consumers. Inadequate diet: 46% low vegetable intake, 44% low fruit intake.	Lack of sustainable funding and monitoring tools; recommendation to integrate into the national strategy. The Need for Screening Integration with Electronic Health Records Ensuring Effective Follow-up for High-Risk Patients.
	**Dyslipidemia Management in Romania**	Focused on lipid control and statin treatment to reduce cardiovascular risk.	2019-ongoing	Public healthcare system, partial out-of-pocket for medication	Recognition of high dyslipidemia prevalence; calls for urgent measures to reduce its impact. Less than 30% of treated patients achieved LDL-C targets, contributing to approximately 260,000 annual CVD-related deaths; 67% of patients with dyslipidemia received statins, indicating undertreatment. Low rate of LDL-C control (<30%); moderate statin use (~67%).	Therapeutic inertia and underdiagnosis; needs better treatment adherence and clinical education. Need for comprehensive national guidelines and increased awareness among healthcare providers.
	**Protocol for treatment of hypercholesterolemia with PCSK9 inhibitors**	National Program, coordinated by the Ministry of Health and financed via the National Health Insurance House, provides access to innovative lipid-lowering therapies for patients after an acute coronary syndrome, at high cardiovascular risk, with familial hypercholesterolemia, or with statin intolerance. It includes PCSK9 inhibitors – Alirocumab (Praluent) and Evolocumab (Repatha). Program has been integrated into a common IT platform, with frequent reporting of quality metrics and outcomes monitoring, to optimize outpatient management.	2015-ongoing	Single National Health Social Insurance Fund	Approximately 8500 patients were given the therapy with PCSK9i in 2024, based on internal Insurance House data analysis (from an eligible population calculated at 237,532 patients).	Extending the number, close to the calculated eligible population.
	**SNAPSHOT epidemiological program**	SNAPSHOT is a multinational, epidemiological, cross-sectional study, with the objectives to investigate assessment of cardiovascular risk and control of blood pressure, LDL-cholesterol, and glycated hemoglobin.	2021-ongoing	Academic Institutions, SEARCH-VASC Centre of Excellence in Research, Servier Pharma	Comparative data between different countries. For Romania, comparative data between different years. It proved a low control of blood pressure (less than 25%) and LDL-cholesterol (less than 12%), and the difficulty of controlling multiple targets in the same patient (in less than 3%), associated with an underestimated cardiovascular risk assessment in half of the patients.	Better implementation of use of SCORE2/SCORE2-OP in clinical practice, and better implementation of guidelines recommendations.
	**SEPHAR (Study for the Evaluation**	SEPHAR is a national epidemiological study conducted by the Romanian Society of Hypertension. It aims to assess the prevalence of hypertension and associated cardiovascular risk factors among the	2005, 2012, 2016	Romanian Society of	Revealed high prevalence of hypertension and other risk factors, underscoring the need for	Continuous monitoring required for updated data.

COUNTRY	Name of the program	Description	Program launch date	Responsible entity	Available results and quality metrics	Limitations / comments
	**of Prevalence of Hypertension and Cardiovascular Risk in Romania)**	adult population in Romania.		Hypertension	improved prevention strategies. Prevalence of hypertension: 45.1%; awareness: 53.8%; treatment in 72.2% of cases; effective control in only 30.8% of cases.	
	**Mission 70 / 2030**	Part of the National Strategy for Management of Cardiovascular and Cerebrovascular Diseases, with the main objective of increasing the control of blood pressure to 70% in 2030.	2025	Romanian Society of Cardiology and Romanian Heart Foundation	Not available.	Ambitious program, including multiple educational activities for physicians and general population.
	**Young Health Programme (YHP) Romania**	Part of a global initiative targeting the prevention of non-communicable diseases among youth, through health education, and promotion of healthy lifestyles.	Initiated in 2019	AstraZeneca, Romanian Heart Foundation	Engaged numerous young individuals through educational sessions, fostering awareness and healthy behaviors.	Sustaining engagement and measuring long-term impact on youth health behaviors.
	**The Heart Walk**	National initiative dedicated to raising awareness and preventing cardiovascular diseases, especially by promoting physical activity and a healthy lifestyle. It is an annual event, usually held in September, on the occasion of World Heart Day (September, the 29th), and consists of a symbolic walk or run along a pre-set, easily accessible route for all ages.	2013-ongoing	Romanian Society of Cardiology and Romanian Heart Foundation in partnership with the Rotary District 2241	40 cities across the country. The routes vary, but they are generally between 1.5 and 3 kilometres long, in central areas or parks.	To transform it in an everyday practice. To promote regular physical activity.
	**Your Heart Speakes to You!**	National campaign to inform and engage opinion leaders (journalists, social media influencers) to pay attention to the signs of angina pectoris. Main goal is to deliver accurate medical information, communicated by physicians, directly through the channels where our target audience gets their information. This represents a modern approach to health education, aligned with public interests and the most effective communication tools available today.	2022-ongoing	Romanian Heart Foundation	Almost 6 million impressions on Facebook, over 40,000 clicks on the website. It reached 1.4 million citizens, which resulted in a successful branding campaign.	To further expand and develop the project.
	**The Heart Gala**	Landmark event with a strategic lobbying component, dedicated to strengthening the relationships between cardiology professionals, pharmaceutical industry leaders, healthcare policymakers, and public and private sector opinion shapers, with a real impact on cardiovascular health policy.	2022, 2025	Romanian Heart Foundation and Romanian Society of Cardiology, under the auspices of the Ministry of Health	Gala provides a prestigious and professional setting for dialogue, partnership, and collective action in support of cardiovascular health in Romania. It emerges as a vital platform for aligning objectives between the public sector, academia, and the pharmaceutical industry.<br>This year's Gala, held on 29th of September (World Heart Day), launched the National Strategy for Management of Cardiovascular and Cerebrovascular Diseases, in the presence of more than 550 participants.	To transform it in an annual lobbying event. To strengthen a national network of cardiovascular health advocates through constructive lobbying and joint initiatives.
**SERBIA**	**Awareness and knowledge of heterozygous familial hypercholesterolemia among Serbian paediatricians**	All registered meeting attendees (a total of 857) received the information of the objective of the study through direct e-mail and were asked to complete the questionnaire using Mentimeter software in the next five days, comprising 6 questions as follows: (1) Is FH a hereditary disease? (2) Are you aware of long-term adverse health effects of FH? (3) Do you know the reference values of cholesterol to establish the diagnosis of FH in children? (4) Do you know the prevalence of FH in general population? (5) Are you familiar with the international guidelines for drug treatment of FH? (6) Do you have cases with FH? We also asked attendees about the duration of their medical practice.	September 2020	Association of Preventive Pediatrics of Serbia	Overall, 91% of participants have knowledge about genetic inheritance of FH, 84.3% were aware of long-term health risks of FH, 77% were familiar with normal cholesterol values in children and 71% knew the FH prevalence in the general population. On the other hand, only 36.8% declared that they were familiar with international guidelines for FH drug treatment and only 26.2% declared to have patients with FH.	There is a substantial lack of practical clinical knowledge among Serbian paediatricians on managing children with FH. Most paediatricians are not aware of the clinical importance of FH in childhood.
	**Prevention of cardiovascular disease: national guidelines for primary care physicians**	The goal of the guidelines is to show general medicine physicians the relevant evidence that will help them in the implementation of preventive measures and therapeutic procedures. In stopping the unfavorable trend of CVD, it should have the greatest importance primary prevention. Prevention of cardiovascular diseases (CVD) involves risk assessment for each patient. General practitioners should implement screening and preventive procedures. Patients who have already had an ischemic event they should be subjected to secondary prevention measures, especially bearing in mind the progress in medical and surgical therapy	2005	Ministry of Health of the Republic Serbia and Serbian Medical Society (supported by European Union and European Agency for	Not available.	

COUNTRY	Name of the program	Description	Program launch date	Responsible entity	Available results and quality metrics	Limitations / comments
				Reconstruction)		
	**Regulation on the national program for the prevention, treatment and control of cardiovascular diseases in the Republic of Serbia until 2020**	Regulation on the national program for the prevention, treatment and control of cardiovascular diseases in the Republic of Serbia until 2020 ("Official Gazette of the RS", No 11/2010). Objectives of the Program: The main goal: Improvement and strengthening of the healthcare system of the Republic of Serbia for better prevention and control of cardiovascular diseases; Specific goals: prevention of risk factors, timely recognition of CVD, improvement of diagnostics, reduction of mortality and disability from CVD, improving the quality of life of patients.	2010-2020	Ministry of Health of the Republic Serbia and Government of the Republic of Serbia	Not available.	
	**Serbia Noncommunicable Diseases Prevention and Control Project (SNDPCP)**	Serbia Noncommunicable Diseases Prevention and Control Project (SNDPCP) is carried out in line with the Loan Agreement signed on August 6, 2024, between the Republic of Serbia and the International Bank for Reconstruction and Development, which, pursuant to the Law on Ratification of the Loan Agreement ("Official Gazette of the RS – International Treaties", No. 9/2024 of December 3, 2024), is implemented by the Ministry of Health and financed through the World Bank loan No. 96040-YF.The objective of the project is to improve health system effectiveness in addressing noncommunicable diseases in Serbia. The project consists of five components: Component 1: Improving provider competence and accountability; Component 2: Increasing availability of services; Component 3: Strengthening quality of public health and clinical services; Component 4: Project management, monitoring and evaluation; Component 5: Contingency emergency response component	2024-2029	Ministry of Health of the Republic of Serbia	Not available.	
**SLOVAKIA**	**MOST Month about Heart Topics**	Project MOST (in English "BRIDGE") was planned like a part of National Cardiovascular Prevention Program. Background for this project raised from findings in cross-sectional studies in Slovakia performed in 1990-2006. Results of these studies clearly shown very low awareness of cardiovascular risk factors, e.g. only 50% of population have information about their blood pressure levels and only about 20% of Slovaks passed lipid levels examination. The main target of the project was to create bridge between scientific knowledge of importance to have very well controlled blood pressure and lipid profile and very bad situation in Slovak population (many people not informed about risk factors or disease, some of them informed, but not satisfactory treated and quite high number are aware of disease but finally were not treated). Slovak Society of Cardiology started this project in September 2007 during two important events - European Society of Cardiology Congress in Vienna and Slovak Society of Cardiology Congress in Bratislava. National campaign in every bigger city resulted in increasing number of people who would like to know their blood pressure, blood glucose and cholesterol level. People were educated by physicians about all important cardiovascular risk factors and level of education seems to have increasing trend up to year 2018. Whole population campaign via PR agencies, media, etc.	2007-2018	Sponsors from various fields of business, media, etc.	Not available. Due to small numbers of examined patients it was possible just to show some positive trends - bigger awareness of risk factors in the population, educational campaign seems to be successful, but again, not properly validated.	Very limited resources, despite professional effort of Slovak Heart Donation and Slovak Society of Cardiology. Very low support by government.
	**IMPAKS**	Implementation of Parameters Control after acute coronary syndrome (ACS). Study performed in 4 Slovak cardiology centres aimed to achieve better control of laboratory parameters and heart function in patients who suffered acute coronary syndrome. Preliminary results showed better control of LDL cholesterol after one year in comparison with existing data from Slovak registry of patients after acute coronary syndrome (SLOVAKS) from previous years. First data showed higher increase of left ventricle ejection fraction. Results have been achieved due to more intensified and more frequent controls after ACS in patients still not optimally treated until they will achieve target level.	2024- in progress	Slovak Society of Cardiology	Study is at the end of recruitment phase. Preliminary results presented at Slovak Society Cardiology congress showed only improved trends - majority of patients still did not reach final visit.	Limitations at the beginning of IMPAKS study were prescription and indication criteria for PCSK9 inhibitors and inclisiran. However, shortly before start of study those limitations were removed for inclisiran only in all patients after ACS not achieving target level of LDL-cholesterol (below 1.4 mmol/l). Ability to prescribe inclisiran helps major part of patients to achieve target levels of LDL cholesterol. Based on existing epidemiological data and data from clinical trials it is possible to expect lowering of cardiovascular morbidity and mortality in this group of patients.
	**MISSION HEALTH CARDIO**	Project is at that time in evaluation by international experts. Expected start of the project after Sep 2025.	2025-2029	European Structural Funds, Slovak Ministry of Health	Not available.	Expected reduction in avoidable cardiovascular deaths and increased number of DALY (mainly in secondary prevention patients).
	**Universal national mandatory**	All children at the age of 11 and 17 years are invited by their primary care paediatricians for total cholesterol measurement from venous blood based on the Methodological Instruction of the Ministry	National paediatric cholesterol	National Health	Exact data are not available. The Slovak National Health Information Center has reported that 94.1%	Data on number of detected and treated children with hypercholesterolemia should be collected. Based on the

COUNTRY	Name of the program	Description	Program launch date	Responsible entity	Available results and quality metrics	Limitations / comments
	**paediatric screening of cholesterol at the age of 11 and 17 years**	of Health 13,010/2004. A threshold level of 188 mg/dL (4.85 mmol/L) is used to stratify care for hypercholesterolemic children. Children with a TC level of 188-209 mg/dL (4.8-5.2 mmol/L) with a negative family history are followed up by a paediatrician. High-risk children and adolescents (those with ≥1 cardiovascular risk factor) are referred to the second level of care (specialists- pediatric cardiologist or endocrinologist) or, if familial hyperlipoproteinemia is suspected, directly to the third level (specialists in the treatment of metabolic disorders in children)	screening (at 11 and 17 years) was initiated in 2004.	System	of 6-14-year-old children and 71.7% of 15-18-year-old adolescents underwent a preventive examination during 2019-2020, screening for cholesterol being a part of this. High rate of participation of mainly 11-years old is therefore expected. Recently published observational study using data of 11-year- old children who underwent TC screening in 23 selected pediatric outpatient clinics between 2017 and 2018 showed 8.9% prevalence of genetically confirmed FH in subset of children with increased total cholesterol (*Raslova K, et al. J Clin Lipidol. 2024; 18(4):e537-e547*).	data, cut-off values for cholesterol can be justified and referral system between primary paediatricians and specialists should be improved.
	**MedPed FH Slovakia**	MedPed (Make early diagnoses to Prevent early deaths in Medical Pedigrees) Familial Hypercholesterolemia (FH) initiative is aimed to identify subjects with FH that are either undiagnosed or inadequately treated and help them treat their disorder by advising them and their doctors on the best possible medical therapies available and to avoid premature manifestation of atherosclerosis. A network of MedPed FH collaborating out-patient departments serve as referring departments for general practitioners and specialists not focused on lipid disorders. MedPed centres diagnose, treat and educate patients with FH and offer cascade screening to family members taking into account 50% risk of FH in first degree relatives.	1997	Srdce rodiny (Heart of family) foundation, Slovak Association of Atherosclerosis, National Health System	More than 2700 patients with suspected FH have been registered and managed in MedPed centres. Data collected on FH patients were used for approval and free of charge cover of PCSK9 inhibitors for selected FH patients in Slovakia. LDL-C treatment goals achievement and reasons for not reaching LDL-C goals in FH patients were evaluated using data from MedPed centers (*Atherosclerosis. 2018; 277:355-361; Atherosclerosis. 2018;277:323-326*) . Data from the initiative have been used for surveillance of familial hypercholesterolaemia both at national and international levels (*Lancet. 2021 ;398(10312):1713-1725; Lancet. 2024 ;403(10421):55-66; Curr Atheroscler Rep. 2019;21(9):36).*	They are still around 80% of expected FH patients not identified in Slovakia. Awareness about FH in primary care physicians and specialists should be improved.
	**Preventive medical examination in adults over 40 years**	Adults > 40 years old have right to absolve every two years within his/her general practician office a preventive medical examination that includes evaluation of cardiovascular risk factors (lipid profile, blood pressure measurement, BMI and smoking status) and ECG examination. SCORE2/SCORE2-OP is also calculated based on the individual risk factors.	2004	National Health System	35-40% of adults underwent the preventive examination in 2015 to 2022 period (*https://www.nczisk.sk/Documents/rocenky/2022/Zdravotnicka_rocenka_Slovenskej_republiky_2022_en.pdf*).	About 60-65% of adults do not participate in the biannual preventive examination. Both positive (e.g. extra benefits for those participating on exams) and negative (e.g. payment contributions by patient for selected medical procedures or treatments for those not participating on exams) motivation approaches can be considered to improve participation rate.
**SLOVENIA**	**Together for Health (previously known as Slovenian National Programme on Primary CVD Prevention)**	The programme provides universal screening for CVD risk factors in adults, followed by measures to reduce overall CVD risk and improve individual risk factors. For nearly 25 years, it has operated with these steps: 1. A basic screening questionnaire to prioritize preventive outpatient visits, 2. Cardiovascular risk assessment during family medicine/GP visits (including cholesterol and glucose tests), 3. Intervention through referrals for additional tests and systematic risk profile changes, focusing on lifestyle counselling and, if needed, medication or other treatments. The chronic noncommunicable diseases (NCD) prevention programme operates in over 1,100 family medicine clinics and 61 health promotion/education centres at primary health centres across Slovenia. Data from preventative visits are collected in uniform computer forms and integrated into GP software. A centralised data collection system supports the national "Registry of Individuals at High CVD Risk". Initially, due to limited resources, the programme targeted high-risk age groups (35-65 for men and 40-70 for women) to ensure preventative care for about 800,000 individuals over five years. From 2002 to 2012, there were 1,044,133 preventative screenings (73.5% of those planned), with 903,428 as first check-ups. Currently, the Together for Health programme at primary care level (https://www.skupajzazdravje.si/en/) supports healthier living for all adults in Slovenia (30+). From 2019-2023, 77,238 healthy people were screened, and 235,677 individuals with NCDs received services from registered nurses at family medicine/GPs. Health centres offer group or individual counselling for lifestyle improvements, including smoking cessation, healthier diets, physical activity, weight loss, and stress management.	The comprehensive primary CVD prevention programme started nationwide in late 2001/early 2002. It was updated and replaced by "Health for All" after 2015 and is currently fully coordinated by the National Institute of Public Health.	National Health Fund. The programme is free for those with compulsory health insurance, including a preventive check-up, group workshops, individual consultations for lifestyle changes and mental health support, and various local health promotion activities.	Individual hard CVD endpoints data is unavailable. However, using standardized death rate data and the IMPACT model approach, approximately 900 lives are saved annually due to primary prevention interventions in comparison to 2002. Notably, premature mortality from ischemic heart disease and stroke has been reduced by over 50%. The Together for Health program effectively improves lifestyles of those at risk of chronic diseases. The program's success is measured through indicators reflecting changes in habits, well-being, and physiological parameters, mainly identifying short-term effects. In 2022, 28% of participants ate vegetables more frequently, and 36% reduced unhealthy snacks. In health-enhancing physical activity, 55% increased balance exercises, while 59% increased muscle-strengthening activities. Over half the participants in type 2 diabetes treatments felt strong enough to manage their diet and exercise. Regarding obesity, 54% of participants in 2022 lowered their body mass index by at least 1 point. In psychoeducation, half the participants believed more in their ability to improve their well-being and felt depression or anxiety could	Patient response rates for CVD risk screening and health services should be improved. Efforts should focus on developing reliable digital tools for systematic data collection and performance analysis.

COUNTRY	Name of the program	Description	Program launch date	Responsible entity	Available results and quality metrics	Limitations / comments
					be overcome.	
	**Integrated care of selected chronic noncommunicable diseases in primary care**	In the Together for Health program, primary care physicians provide structured and coordinated care for chronic noncommunicable diseases (NCDs). This includes personalized treatment plans, enhanced secondary prevention, and quicker access to specialists. Care covers NCDs like CVD (hypertension and ischemic heart disease), diabetes, COPD, asthma, osteoporosis, prostatic disease, and depression. Physicians conduct regular control visits to assess and adjust care plans, supported by trained registered nurses. Care follows guidelines set by specialist societies, with specialist consultations available via referrals or remote means.	2015	National Health Fund	Data on individual CVD endpoints are unavailable, and data on CVD risk factors management are incomplete.	The programme is implemented in most family medicine/GP clinics in Slovenia, but implementation and patient coverage vary due to a shortage of primary care physicians. Timely specialist consultations are often difficult to provide. Reliable data on the programme's effectiveness and efficiency is lacking due to unsystematic data collection. However, the upcoming introduction of EHRs is expected to improve the situation.
	**Comprehensive outpatient cardiac rehabilitation programmes**	Before 2017, cardiac rehabilitation (CR) in Slovenia was under-utilised and limited mainly to short-term residential programmes. To expand CR content and provision, key stakeholders (including the Slovenian Society of Cardiology, the Health Insurance Institute of Slovenia, the National Institute of Public Health, and the Slovenian Forum for Cardiovascular Disease Prevention) proposed the set-up of comprehensive outpatient CR centres affiliated with each regional hospital in the country. Until now, seven regional centres were established to provide comprehensive multidisciplinary outpatient CR. Comprehensive outpatient cardiac rehabilitation (CR) programmes provide CR over a 3-month period, comprising: i) intake visit with comprehensive cardiovascular assessment (including initial exercise testing and laboratory analyses), ii) 36 exercise training sessions under physical/exercise therapist supervision, iii) risk factor management (including specialist blood pressure, lipids and diabetes management, and access to smoking cessation and weight management programmes), iv) secondary preventive medication management and monitoring (under cardiologist supervision), v) nurse-led patient education, nutritional counselling and health promotion (three group sessions and individual counselling as needed), vi) screening for mood disorders (with priority access to mental health consultation), and vii) final assessment (including exercise testing, laboratory analysis, echocardiography, and structured transition to long-time care, including referral to 'phase III' community programmes). Exercise sessions are 2-3 times per week, featuring 60 minutes of moderate aerobic and low-intensity resistance training.	Hospital-affiliated centre-based outpatient CR programmes began in January 2017, including facility set-up, personnel training, and referral pathways for myocardial infarction patients, recommending CR intake within 30 days post-discharge. Setup finished in 6 months under the Centre for Preventive Cardiology, University Medical Centre Ljubljana.	National Health Fund. Program set-up reimbursement covered cardiology supervision, nurse-led lifestyle intervention, exercise sessions, intake, exercise testing, lab analyses, echocardiography, and necessary referrals (e.g., psychologist, occupational consultations). Full reimbursement required completing 36 sessions, exclusive of residential short-term CR participation.	Establishing dedicated regional comprehensive outpatient centers enhances CR uptake post-myocardial infarction. The proportion of patients participating in CR increased from 25% before the introduction of comprehensive outpatient CR programmes in 2016 to 40% by 2020. Participation in outpatient or short-term residential CR leads to a 42% and 21% reduction in death and cardiovascular hospitalizations, respectively, with mortality decreasing by 46% and 41%. A five-year interim analysis (2017 -- 2021) indicates outpatient CR is more effective in reducing cardiovascular hospitalizations.	Until now, seven regional centres providing comprehensive outpatient CR were established in Slovenia, while the aim is to assure these services nationwide. The participation rate of post-MI patients should be increased. The data on performance and quality indicators are collected within the National CR Registry. It is our plan to assure the completeness of data by joining the EuroHeart Registry platform in the near future.
	**Universal screening on FH at the age of 5**	Slovenia (population 2 million) started universal screening for hypercholesterolemia in 5-year-old children in 1995. Screening was gradually implemented throughout the whole country, reaching an estimated and sustained majority of 5-year-old children (>90% of approximately 20,000) during 2013. Measurement of fasting serum TC is part of the national programmed routine examination of 5-year-old children by primary paediatricians (Slovenia national universal screening for hypercholesterolemia). According to national guidelines, participants with either a serum TC level of >6 mmol/l (231.7 mg/dl) without a family history of premature cardiovascular complications or with a TC level of >5 mmol/l (193.1 mg/dl) with a family history positive for premature cardiovascular complications are referred after a consultation with their parents to a tertiary pediatric outpatient clinic. Data for participants' family histories are obtained in direct structured conversations with the parents. Participants' weight, body mass index, serum lipid profile (TC, LDL, high-density cholesterol [HDL], triglyceride), and whole-blood samples for targeted, next-generation sequencing (NGS) are obtained. Simon Broome Register criteria are used for assessment of family history. Positive family history is defined as MI before 50 years of age in any second-degree relative or before 60 years of age in any first-degree relative or as TC >7.5 mmol/l (289.6 mg/dl) in any first- or second-degree relative (first-degree relation included parent, offspring or sibling; second-degree relation included grandparent, grandchild, nephew, niece, or half-sibling). Written informed consent is obtained from all parents or legal guardians. Excluding secondary dyslipidemia is crucial in diagnostics. Patients should receive cardiology care post-pediatric clinic.	1995 (approx. 30 years)	National Health Fund	For every 1000 children screened, 2.3 are identified with positive FH results and 3.4 with possible FH. The prevalence is 1/273 for possible FH, 1/409 for confirmed FH, and 1/297,000 for homozygous FH. Most children with FH are now diagnosed each generation. Positive tests enable cascade FH genetic testing for parents with high cholesterol. The Slovenian Heart Foundation reports that diagnosis rates increased from 1% to 20% in 15 years. Direct costs for new confirmed FH cases are about $1,015.	Screening was gradually implemented nationwide during the programmed visit of all children aged five years at the primary care paediatrician, and as shown by the Slovenian national FH registry, the programme now reaches approximately 91% of the population of around 20,000 children each year. The main limitation is that currently still approx. 10% of children do not participate in the 5-year old's examination (despite it is obligatory).
